# A boundary-integral representation for biphasic mixture theory, with application to the post-capillary glycocalyx

**DOI:** 10.1098/rspa.2014.0955

**Published:** 2015-07-08

**Authors:** P. P. Sumets, J. E. Cater, D. S. Long, R. J. Clarke

**Affiliations:** 1 Department of Engineering Science, University of Auckland, Auckland 1142, New Zealand; 2 Auckland Bioengineering Institute, University of Auckland, Auckland 1142, New Zealand

**Keywords:** biphasic mixture theory, boundary element methods, endothelial glycocalyx layer

## Abstract

We describe a new boundary-integral representation for biphasic mixture theory, which allows us to efficiently solve certain elastohydrodynamic–mobility problems using boundary element methods. We apply this formulation to model the motion of a rigid particle through a microtube which has non-uniform wall shape, is filled with a viscous Newtonian fluid, and is lined with a thin poroelastic layer. This is relevant to scenarios such as the transport of small rigid cells (such as neutrophils) through microvessels that are lined with an endothelial glycocalyx layer (EGL). In this context, we examine the impact of geometry upon some recently reported phenomena, including the creation of viscous eddies, fluid flux into the EGL, as well as the role of the EGL in transmitting mechanical signals to the underlying endothelial cells.

## 1. Introduction

Biphasic mixture theory is commonly used to model linearly poroelastic materials, including cartilage [1], and the brush-like layer that lines the microvasculature, namely the endothelial glycocalyx layer (EGL) [2]. It has long been known how to represent the fluid phase of these biphasic mixture theory models in boundary-integral form [3]. This is a convenient formulation as it describes the flow entirely in terms of quantities defined on flow surfaces, thereby effectively reducing the dimensionality of the problem. However, reformulating the solid phase into boundary-integral form is hampered by the appearance of volume integrals that arise from the coupling between the fluid and solid phases. Here, we demonstrate how these volume integrals can be recast into surface integrals, to yield a boundary-integral formulation for the full poroelastic dynamics. In doing so, we open up biphasic mixture theory models to a boundary element method (BEM) numerical treatment.

Although we present a general purpose boundary-integral formulation for linearly poroelastic materials, our immediate interest in this problem is motivated by the microvasculature, and our choice of physical parameters is informed by this physical regime. The walls of blood vessels are composed of endothelial cells which have non-uniform shape, and which can be coated with a layer consisting of a mixture of macro-molecules. In the literature, this brush-like layer is referred to as the EGL, or sometimes the endothelial surface layer. This layer has a gel-like structure and comprises proteoglycans, glycosaminoglycans, glycoproteins and absorbed plasma proteins [4]. The EGL is believed to serve a number of functions, including mechanotransmission of fluid shear stress (FSS) to the endothelial actin cortical cytoskeleton (degradation of the EGL is seen to be correlated with the endothelial cells becoming less likely to align with the flow, i.e. remodel [5]), a modulator of permeability in the transcapillary exchange of water, and as a regulator in the inflammatory response where it is believed to play a role in the leucocyte adhesion cascade [6]. In addition, the results obtained by Vink *et al.* [7] suggest that, along with other functions, the EGL plays a major role in providing vessels with an anti-adhesive inner lining. As such, the interplay between the EGL, the flow of blood plasma within the lumen and cells within the plasma is expected to be an important one for cardiovascular health.

The motion of cells through the microvasculature is a topic that has received much previous theoretical treatment. Some models have considered rigid cells passing through a straight vessel in the absence of an EGL [8,9]. When the cell is allowed to deform, as do red blood cells (RBCs), cell-depleted regions form adjacent to the vessel walls, and an accompanying drop in the apparent viscosity of the fluid is observed [10]. The role of a rigid, but porous, EGL in the migration of deformable cells from rigid surfaces has recently been considered, where it is suggested that the EGL may act to reduce the thickness of the depletion layer [11]. Boundary-integral formulations already exist that can describe these EGL-free or rigid-EGL scenarios [12,13].

However, the EGL is deformable, and some previous studies have accounted for its poroelastic behaviour using biphasic mixture theory. In a wavy-walled vessel, and in the absence of any cells, it has been demonstrated how the flow can separate and form a recirculation region which may, in turn, influence molecular transport and cellular response [14]. Other studies have adopted a similar approach to consider the passage through the lumen of rigid cells [2], EGL-coated cells [15] and cells which can undergo large deformations (e.g. RBCs) [16–19]. These studies have shown that the poroelastic layer can significantly affect the apparent viscosity of the flow, and that hydrodynamic forces can perhaps explain an observed exclusion of RBCs from the EGL under flow [18,20]. There is also the suggestion that the presence of the compliant EGL can reduce the FSSs exerted upon a RBC as it passes through non-uniformly shaped microvessels [19]. The EGL is also predicted to suppress FSSs on the endothelial wall, which seems at odds with its role as a transducer of mechanical stresses. It is now hypothesized that a significant portion of the stress is actually carried through the solid phase of the EGL.

These previous biphasic mixture theory models of EGL-lined microvessels have typically adopted a lubrication theory approximation, which places certain geometrical constraints upon the model (i.e. a long-wavelength analysis). By implementing our boundary-integral representation for biphasic mixture theory, we are now able to consider the aforementioned effects in a more general setting. Special attention is paid to the effect of geometry upon the system as a whole and the interaction between the flow, particle and poroelastic layer.

In §2, we show how the governing equations for biphasic mixture theory can be recast into boundary-integral form. The results we present in §3 for the two-dimensional case show how wall shape and the presence of a rigid cell affect the flow and solid displacements of the EGL. We draw some conclusions from these observations in §4.

## 2. Formulation

### (a) Geometry

**Figure 1.**
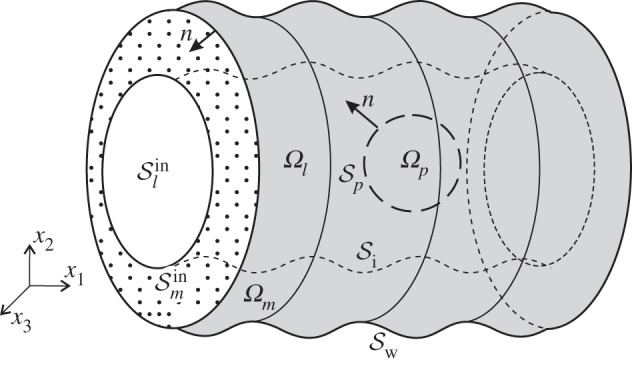
A diagram of the geometry of the microtube containing a rigid cell, and lined with a poroelastic layer.

Our geometry consists of a lumen of radius H*, and volume *Ω*_*l*_, through which an incompressible Newtonian fluid can flow unhindered, as well as a poroelastic layer, *Ω*_*m*_, which is attached to the vessel walls. The lumen contains a rigid particle, *Ω*_*p*_, with surface $S_{p}$. The lumen and the poroleastic layer are bounded by the surfaces, $S_{l}$ and $S_{m}$, respectively. The tube has a rigid wall denoted by $S_{w}$. Surface $S_{i}$ forms the interface between the lumen and the poroelastic layer. Denoting inlet and outlet surfaces as $S^{\mathrm{in}}\;\mathrm{and}\;S^{\mathrm{out}}$, we have $S_{m}=S_{m}^{\mathrm{in}}\cup S_{m}^{\mathrm{out}}\cup S_{i}\cup S_{w}$ and $S_{l}=S_{l}^{\mathrm{in}}\cup S_{l}^{\mathrm{out}}\cup S_{i}$. See figure 1 for a diagram of the geometry.

The intrinsic masses and the volumes occupied by the fluid and solid phases in the poroelastic layer are *m*_f_,*V*_f_ and *m*_s_,*V*_s_, respectively. The total volume of the poroelastic layer is therefore *V* =*V*_f_+*V*_s_. Introducing the volume fractions for each phase as *ϕ*_f_=*V*_f_/*V* and *ϕ*_s_=*V*_s_/*V* , we can define two partial densities for each phase: *ρ*_f_=*ϕ*_f_*m*_f_/*V*_f_ and *ρ*_s_=*ϕ*_s_*m*_s_/*V*_s_. The requirement that there are no voids in the poroelastic layer necessitates that *ϕ*_f_+*ϕ*_s_=1.

There is a flow with characteristic speed, *V* *, through the lumen, which in turn drives flows and elastic deformations within the poroelastic layer, as well as transporting a rigid particle through the lumen.

### (b) Particle motility

We assume that the particle moves as an impermeable rigid object (as might be largely the case for white blood cells, for example), and hence the instantaneous particle velocity, V*_p_, can be written as

2.1 $${\text{V}}_{p}^{*}={\text{W}}^{*}+\omega_{p}^{*}\text{k}\times ({\text{x}}^{*}-{\text{x}}_{c}^{*}),$$

where x* is a point on the surface of the particle, x*_c_ is its centre and ***k*** is the axis of rotation (asterisks denote dimensional quantities). The particle translational velocity, W*, and angular velocity, ω*_p_, are initially unknown. We non-dimensionalize using

2.2 $$\text{x}=\frac{{\text{x}}^{*}}{H^{*}},\;\text{W}=\frac{{\text{W}}^{*}}{V^{*}}\;\mathrm{and}\;\omega_{p}=\frac{\omega_{p}^{*}H^{*}}{V^{*}},$$

which gives

2.3 $${\text{V}}_{p}=\text{W}+\omega_{p}\text{k}\times (\text{x}-{\text{x}}_{c}).$$

In the absence of inertia (due to the small scale of the flow) and external forces or torques, the particle must be both force and torque free, i.e.

2.4 $$\int_{S_{p}}{\text{f}}_{p}\;ds(\text{x})=0,\;\int_{S_{p}}(\text{x}-{\text{x}}_{c})\times {\text{f}}_{p}\;ds(\text{x})=0,$$

where ***f***_p_ is the traction on the particle surface (see §2c).

### (c) Hydrodynamics in the lumen

As we consider steady flows through very small tubes, such as those that might typify post-capillary venules or capillaries, the governing flow equations in the lumen are the incompressible Stokes flow equations

2.5 $$\mu_{f}\nabla^{2}{\text{v}}^{*}=\nabla P^{*},\;\nabla \cdot {\text{v}}^{*}=0,$$

where ${\text{ v}}^{*}=(v_{1}^{*},v_{2}^{*},v_{3}^{*})$ and P* are the flow velocities and pressure, and *μ*_f_ is the dynamic viscosity of the fluid. The Cauchy stress tensor for the fluid has the form σ*=−P*I+(∇v*+(∇v*)^T^) (superscript T denotes transpose). Non-dimensionalizing according to

2.6 $$\text{x}=\frac{{\text{x}}^{*}}{H^{*}},\;\text{v}=\frac{{\text{v}}^{*}}{V^{*}},\;P=\frac{P^{*}H^{*}}{\left(\mu_{f}V^{*}\right)}\;\mathrm{and}\;\sigma =\frac{\sigma^{*}H^{*}}{\left(\mu_{f}V^{*}\right)}$$

yields

2.7 $$\nabla^{2}\text{v}=\nabla P,\;\nabla \cdot \text{v}=0.$$

This can be recast into the following boundary-integral form [3]:

2.8 $$\begin{array}{rl} & -\int_{S_{l}}f_{j}(\text{x})G_{ij}(\text{x},{\text{x}}_{0}^{\prime })\;ds(\text{x})+{⨍\;-}_{S_{l}}v_{j}(\text{x})T_{ijk}(\text{x},{\text{x}}_{0}^{\prime })n_{k}(\text{x})\;ds(\text{x})\\ & \;+W_{j}{⨍\;-}_{S_{p}}T_{ijk}(\text{x},{\text{x}}_{0}^{\prime })n_{k}(\text{x})\;ds(\text{x})+\omega_{p}{⨍\;-}_{S_{p}}\left(\text{k}\times {\left(\text{x}-{\text{x}}_{c})\right)}_{j}T_{ijk}(\text{x},{\text{x}}_{0}^{\prime })n_{k}(\text{x})\;ds(\text{x}\right)\\ & \;=c_{f}\left\{\begin{array}{l} v_{i}({\text{x}}_{0}^{\prime }),\;{\text{x}}_{0}^{\prime }\in S_{l}\\ W_{i}+\omega_{p}{\left(\text{k}\times ({\text{x}}_{0}^{\prime }-{\text{x}}_{c})\right)}_{i},\;{\text{x}}_{0}^{\prime }\in S_{p}, \end{array}\right. \end{array}$$

*c*_*f*_=2*π* (*i*,*j*,*k*=1,2) for the two-dimensional case, and *c*_*f*_=4*π* (*i*,*j*,*k*=1,2,3) for three-dimensional flow. The integrals involving the Stokeslet *G*_*ij*_ are referred to as the single-layer potentials, whereas the second, third and fourth integrals involving *T*_*ijk*_, the associated stress tensor, are only defined in a Cauchy principal value sense, and referred to as the double-layer potentials (similar terminology applies for the boundary-integral representations which follow). Details of the exact form of these tensors are given in the electronic supplementary material, S1. Here, ***f***=***σ***⋅***n*** is the surface traction on the Stokes flow surfaces and ***n*** is the inward normal vector.

### (d) Elastohydrodynamics in the poroelastic layer

The poroelastic layer consists of a solid phase which is linearly elastic, and a fluid phase that obeys the porous flow equations. In what follows, the subscript *s* refers to the solid phase, while subscript *f* refers to the fluid phase. Following earlier work [2,14], we model the poroelastic layer using biphasic mixture theory [21–23] and make the assumption that each phase is incompressible, has the same density, is homogeneous and has negligible inertia.

From incompressibility, we have the mass conservation equation

2.9 $$\nabla \cdot (\phi_{f}{\text{w}}^{*}+\phi_{s}{\text{v}}_{s}^{*})=0,$$

where w* is the velocity of the fluid phase and v*_s_ is the velocity vector of the solid phase.

The momentum equations then take the form [22]

2.10 $$\nabla \cdot (\phi_{f}\sigma_{f}^{*})=\pi^{*}$$

and

2.11 $$\nabla \cdot (\phi_{s}\sigma_{s}^{*})=-\pi^{*}$$

(note that ∇⋅(*ϕ*_f_σ*_f_+*ϕ*_s_σ*_s_)=0) where σ*_f_ and σ*_s_ are the intrinsic Cauchy stress tensors for the fluid and solid phases, respectively, and π* is the momentum transfer tensor which expresses the force coupling due to the interaction between the two phases, which we define below.

For small strains (linear elastic theory), the constitutive equations for each phase are [22,24]

2.12 $$\sigma_{f}^{*}=-p^{*}\text{I}+\mu_{f}{\left(\nabla {\text{w}}^{*}+(\nabla {\text{w}}^{*}\right)}^{T})$$

and

2.13 $$\sigma_{s}^{*}=-p^{*}\text{I}+\mu_{s}(\nabla {\text{u}}^{*}+{\left(\nabla {\text{u}}^{*}\right)}^{T})+\lambda_{s}(\nabla \cdot {\text{u}}^{*})\text{I},$$

where p* is the flow pressure, u* is the displacement vector of the solid phase, *μ*_f_ is the dynamic viscosity of the fluid (which we assume to be the same as that in the lumen), and λ_s_ and *μ*_s_ are the Lame constants of the solid phase. The total stress is then

2.14 $$\begin{array}{rl} \Gamma^{*} & =\phi_{f}\sigma_{f}^{*}+\phi_{s}\sigma_{s}^{*}=-p^{*}\text{I}+\phi_{f}\mu_{f}(\nabla {\text{w}}^{*}+{\left(\nabla {\text{w}}^{*}\right)}^{T})\\ & \;+\phi_{s}\mu_{s}(\nabla {\text{u}}^{*}+{\left(\nabla {\text{u}}^{*}\right)}^{T})+\phi_{s}\lambda_{s}(\nabla \cdot {\text{u}}^{*})\text{I}. \end{array}$$

It can be seen that the total stress tensor represents the sum of fluid and elastic stress tensors with rescaled material constants, corresponding to the partial stress tensors.

Following [2,14], momentum transfer is given by

2.15 $$\pi^{*}=K^{*}({\text{w}}^{*}-{\text{v}}_{s}^{*}),$$

where K* is the hydraulic resistivity of the biphasic mixture. As the solid phase is attached to an immovable solid wall, at steady state, in the Cartesian coordinates fixed on the tube, the solid velocities are zero. Hence,

2.16 $$\pi^{*}=K^{*}{\text{w}}^{*}.$$

#### (i) Fluid phase

Conservation of mass (assuming v*_s_=***0***) dictates that

2.17 $$\nabla \cdot {\text{w}}^{*}=0.$$

With regards to momentum conservation, upon substituting (2.12) and (2.16) into (2.10), we obtain the Brinkman-type equation for the fluid phase [25],

2.18 $$\phi_{f}\mu \nabla^{2}{\text{w}}^{*}=\phi_{f}\nabla p^{*}+K^{*}{\text{w}}^{*},\;\nabla \cdot {\text{w}}^{*}=0.$$

We note that, owing to v*_s_=***0***, we can solve (2.18) for the fluid phase before determining the elastic deformation of the solid phase. Non-dimensionalizing according to

2.19 $$\text{x}=\frac{{\text{x}}^{*}}{H^{*}},\;\text{w}=\frac{{\text{w}}^{*}}{V^{*}},\;p=\frac{p^{*}H^{*}}{\left(\mu_{f}V^{*}\right)}\;\mathrm{and}\;\sigma_{f}=\frac{\sigma_{f}^{*}H^{*}}{\left(\mu_{f}V^{*}\right)}$$

gives

2.20 $$\nabla^{2}\text{w}=\nabla p+\chi \text{w},\;\nabla \cdot \text{w}=0,$$

where *χ*=K*H*^2^/(*ϕ*_f_*μ*_f_) is a measure of porous resistance. Brinkman flow also has a well-known boundary-integral representation (equivalent to that for oscillatory Stokes flow [3])

2.21 $$c_{f}w_{i}({\text{x}}_{0})=-\int_{S_{m}}g_{j}(\text{x})M_{ij}(\text{x},{\text{x}}_{0})\;ds(\text{x})+{⨍\;-}_{S_{m}}w_{j}(\text{x})R_{ijk}(\text{x},{\text{x}}_{0})n_{k}(\text{x})\;ds(\text{x}),$$

where *M*_*ij*_ and *R*_*ijk*_ are the free-space singularity solutions to Brinkman’s equation (see the electronic supplementary material, S1). The coefficient *c*_*f*_ here is as defined for the boundary-integral representation for Stokes flow within the lumen (2.8), and $\text{ g}=\sigma_{f}\cdot \text{ n}(S_{m})$ is the surface traction due to flow in the fluid phase.

#### (ii) Solid phase

Substituting (2.13) and (2.16) into (2.11) yields the momentum conservation equations for the solid phase

2.22 $$\phi_{s}(\lambda_{s}+\mu_{s})\nabla (\nabla \cdot {\text{u}}^{*})+\phi_{s}\mu_{s}\nabla^{2}{\text{u}}^{*}=\phi_{s}\nabla p^{*}-K^{*}{\text{w}}^{*}.$$

Hence, the solid phase is governed by the steady Navier equation with two forcing terms.

Non-dimensionalizing using

2.23 $$\text{x}=\frac{{\text{x}}^{*}}{H^{*}},\;\text{u}=\frac{{\text{u}}^{*}\phi \mu_{s}}{\left(V^{*}\mu_{f}\right)}\;\mathrm{and}\;\sigma_{s}=\frac{\sigma_{s}^{*}\phi H^{*}}{\left(\mu_{f}V^{*}\right)}$$

gives

2.24 $$\frac{1}{1-2\nu }\nabla (\nabla \cdot \text{u})+\nabla^{2}\text{u}=\phi \nabla p-\chi \text{w},$$

where *ϕ*=*ϕ*_s_/*ϕ*_f_ and *ν*=λ_s_/2(λ_s_+*μ*_s_) is Poisson’s ratio. Following the usual procedure for writing the Navier equation in boundary-integral form, we can use the Maxwell–Betti reciprocal relation to obtain the following integral form for the behaviour of the solid phase [26]:

2.25 $$\begin{array}{rl} c_{s}u_{i}({\text{x}}_{0}) & =-\int_{S_{m}}h_{j}(\text{x})S_{ij}(\text{x},{\text{x}}_{0})\;ds(\text{x})+{⨍\;-}_{S_{m}}u_{j}(\text{x})K_{ijk}(\text{x},{\text{x}}_{0})n_{k}(\text{x})\;ds(\text{x})\\ & \;-\phi \int_{\Omega_{m}}p_{,j}(\text{x})S_{ij}(\text{x},{\text{x}}_{0})\;d\Omega +\chi \int_{\Omega_{m}}w_{j}(\text{x})S_{ij}(\text{x},{\text{x}}_{0})\;d\Omega , \end{array}$$

with *c*_s_=4*π*(1−*ν*) or *c*_s_=8*π*(1−*ν*) in two and three dimensions, respectively. Here, *p*_,*j*_ stands for the partial derivative with respect to the *x*_*j*_ coordinate, and $\text{ h}=(\sigma^{s}+\phi p\text{ I})\cdot \text{ n}(S_{m})$ is the traction vector for the elastic part of the solid phase. Also *S*_*ij*_ and *K*_*ijk*_ are the Green’s function and fundamental stress tensor for isotropic linear elasticity (i.e. Kelvin solutions; see the electronic supplementary material, S1). This is not yet in boundary-integral form, due to the volume integrals of the two forcing terms. We shall now show how these can also be converted into surface integrals. We consider each forcing term in turn.

*Pressure forcing*. To convert the volume integral involving pressure gradients into a surface integral, we use divergence theorem and Green’s identities along with the property of fundamental solutions (see details in appendix Aa),

2.26 $$\int_{\Omega_{m}}p_{,j}S_{ij}\;d\Omega =(1-2\nu )\int_{S_{m}}{\hat{x}}_{i}\beta \frac{\partial p}{\partial n}\;ds+2(1-\nu )\int_{S_{m}}2p\beta \delta_{ik}n_{k}-p\frac{\partial ({\hat{x}}_{i}\beta )}{\partial n}\;ds,$$

where ${\hat{x}}_{i}={\left(\text{ x}-{\text{ x}}_{0}\right)}_{i},\;r=|\hat{\text{ x}}|$, and β=ln⁡r or *β*=−1/*r* in two and three dimensions, respectively. In order to use identity (2.26), we need to know both the pressure and its normal derivative, ∂*p*/∂*n*, on the boundary. The boundary-integral flow representation (2.21) yields only flow velocities and tractions, from which surface pressures can be determined from the following boundary-integral relation [27]:

2.27 $$c_{f}p({\text{x}}_{0})=-{⨍\;-}_{S_{m}}g_{i}(\text{x})Q_{i}(\text{x},{\text{x}}_{0})\;ds(\text{x})+\underset{S_{m}}{⨍\;=}w_{i}(\text{x})L_{ik}(\text{x},{\text{x}}_{0})n_{k}(\text{x})\;ds(\text{x})$$

(singularity solutions *Q*_i_ and *L*_*ik*_ are given in the electronic supplementary material, S1). The first integral is a Cauchy principal value integral, and the second a hyper-singular integral that must be regularized for numerical treatment (see appendix Aa for details). Consequently,

2.28 $$\begin{array}{rl} c_{f}p({\text{x}}_{0}) & =-{⨍\;-}_{S_{m}}g_{i}(\text{x})Q_{i}(\text{x},{\text{x}}_{0})\;ds(\text{x})\\ & \;+{⨍\;-}_{S_{m}}\left(w_{i}(\text{x})-w_{i}({\text{x}}_{0}))L_{ik}(\text{x},{\text{x}}_{0})n_{k}(\text{x})\;ds(\text{x}\right)-2\chi w_{i}({\text{x}}_{0})\int_{S_{m}}n_{i}\beta \;ds(\text{x}). \end{array}$$

Once surface pressure is known, its normal gradient can be determined through Green’s third identity,

2.29 $$\int_{S_{m}}\beta \frac{\partial p}{\partial n}\;ds(\text{x})=\frac{c_{f}}{2}p({\text{x}}_{0})+{⨍\;-}_{S_{m}}p(\text{x})\frac{\partial \beta }{\partial n}\;ds(\text{x}),\;{\text{x}}_{0}\in S_{m}.$$

*Momentum transfer*. We now turn to the volume integral in (2.25) that stems from the momentum transfer between phases. Our approach involves consideration of the flow within the fluid phase (2.21)

2.30 $$\nabla \cdot \sigma_{f}=\text{F},$$

where *F*_*j*_=*χw*_*j*_, alongside a second complementary flow defined by

2.31 $$\nabla \cdot \sigma^{B_{i}}={\text{F}}^{B_{i}},$$

where $F_{j}^{B_{i}}=\chi v_{j}^{B_{i}}+S_{ij}$, *S*_*ij*_ is Green’s function for linear elasticity, and ***σ***^*B*_*i*_^, ***v***^*B*_*i*_^ are the Cauchy stress tensor and flow velocity for the complementary flow.

We then apply the Lorentz reciprocal relation to flows (***σ***_f_,***w***) and (***σ***^*B*_*i*_^,***v***^*B*_*i*_^), integrate over the flow domain and apply the divergence theorem to obtain [3]

2.32 $$\int_{S_{m}}g_{j}v_{j}^{B_{i}}\;ds(\text{x})+\int_{\Omega_{m}}F_{j}v_{j}^{B_{i}}\;d\Omega =\int_{S_{m}}g_{j}^{B_{i}}w_{j}\;ds(\text{x})+\int_{\Omega_{m}}F_{j}^{B_{i}}w_{j}\;d\Omega ,$$

where ***g***^*B*_*i*_^=***σ***^*B*_*i*_^⋅***n***. It can be seen that, after substitution of the expressions for ***F*** and ***F***^*B*_*i*_^ into (2.32), we obtain the volume integral in terms of boundary integrals

2.33 $$\int_{\Omega_{m}}w_{j}S_{ij}\;d\Omega =-\int_{S_{m}}g_{j}^{B_{i}}w_{j}\;ds(\text{x})+\int_{S_{m}}g_{j}v_{j}^{B_{i}}\;ds(\text{x}),\;{\text{x}}_{0}\in \Omega_{m}.$$

Hence, the problem now reduces to finding a flow which satisfies (2.31), and which is given by

2.34 $$v_{j}^{B_{i}}=\frac{2-2\nu }{\chi }\left[\frac{1}{r}(A(\eta )-1)\delta_{ij}+\frac{{\hat{x}}_{i}{\hat{x}}_{j}}{r^{3}}(B(\eta )-1)\right],$$

where

2.35 $$A(\eta )=2\;e^{-\eta }\left(1+\frac{1}{\eta }+\frac{1}{\eta^{2}}\right)-\frac{2}{\eta^{2}},\;B(\eta )=-2\;e^{-\eta }\left(1+\frac{3}{\eta }+\frac{3}{\eta^{2}}\right)+\frac{6}{\eta^{2}}$$

and $\eta =\sqrt{\chi }r$ (see appendix Ab for details).

*Boundary-integral representation*. We arrive at the final boundary-integral representation for the solid phase

2.36 $$\begin{array}{rl} c_{s}u_{i}({\text{x}}_{0}) & =-\int_{S_{m}}h_{j}(\text{x})S_{ij}(\text{x},{\text{x}}_{0})\;ds(\text{x})+{⨍\;-}_{S_{m}}u_{j}(\text{x})K_{ijk}(\text{x},{\text{x}}_{0})n_{k}(\text{x})\;ds(\text{x})\\ & \;-\phi (1-2\nu )\int_{S_{m}}q(\text{x}){\left(\text{x}-{\text{x}}_{0}\right)}_{i}\beta (\text{x},{\text{x}}_{0})\;ds(\text{x})\\ & \;-2\phi (1-\nu )\int_{S_{m}}p(\text{x})\left(2\beta (\text{x},{\text{x}}_{0})\delta_{ik}n_{k}(\text{x})-\frac{\partial ({\left(\text{x}-{\text{x}}_{0}\right)}_{i}\beta (\text{x},{\text{x}}_{0}))}{\partial n}\right)ds(\text{x})\\ & \;+\chi \left(-\int_{S_{m}}w_{j}(\text{x})g_{j}^{B_{i}}(\text{x},{\text{x}}_{0})\;ds(\text{x})+\int_{S_{m}}g_{j}(\text{x})v_{j}^{B_{i}}(\text{x},{\text{x}}_{0})\;ds(\text{x})\right), \end{array}$$

where *q*(***x***)=∂*p*/∂*n*.

### (e) Boundary conditions

As we assume that the particle is impermeable, with a no-slip surface, $S_{p}$, we have

2.37 $$\text{v}(\text{x})=V_{p},\;\text{x}\in S_{p}.$$

The vessel wall, $S_{w}$, is assumed to be rigid and impermeable, hence we have

2.38 $$\text{w}(\text{x})=\text{u}(\text{x})=0,\;\text{x}\in S_{w}.$$

On the inlet and outlet surfaces, we prescribe the analytical solutions for displacement and velocity corresponding to flow through a straight-walled, poroelastic-lined tube, ***U***_0_,***W***_0_,***V***_0_ (see the electronic supplementary material, S2), i.e.

2.39 $$\text{v}(\text{x})={\text{V}}_{0}(\text{x}),\;\text{w}(\text{x})={\text{W}}_{0}(\text{x}),\;\text{u}(\text{x})={\text{U}}_{0}(\text{x}),\;\text{x}\in S_{m}^{\mathrm{in}},S_{m}^{\mathrm{out}},S_{l}^{\mathrm{in}},S_{l}^{\mathrm{out}}.$$

As there is no inertia in the flow, we expect entry and exit development effects to be localized, typically scaling with tube radius.

As we assume small-strain elasticity, in keeping with earlier models, all boundary conditions on the interfaces can be applied at their undeformed locations, $S_{i}$. The first condition on the interfaces is continuity of flow velocity (accounting for the fact that we assume ***v***_s_=***0***, i.e. no elastic velocities)

2.40 $$\phi_{f}\text{w}(\text{x})=\text{v}(\text{x}),\;v\in S_{i}.$$

The second interface boundary condition is continuity of traction [28]

2.41 $$\text{n}\cdot \Gamma (\text{x})=\text{n}\cdot \sigma (\text{x}),\;\text{x}\in S_{i},$$

where ***n*** is a unit normal to the boundary and ***Γ***=*ϕ*_f_***σ***_f_+*ϕ*_s_(***σ***_s_/*ϕ*) is the total stress in the poroelastic material. The proportion of the total stress in the porous medium borne by each phase is proportional to its volume fraction, hence

2.42 $$\frac{\phi_{s}}{\phi }(\text{h}(\text{x})-\phi p\text{I}\cdot \text{n})=\phi_{s}\text{f}(\text{x}),\;\text{x}\in S_{i}$$

and

2.43 $$\phi_{f}\text{g}(\text{x})=\phi_{f}\text{f}(\text{x}),\;\text{x}\in S_{i}$$

(recalling that *ϕ*_s_+*ϕ*_f_=1).

## 3. Results

**Figure 2.**
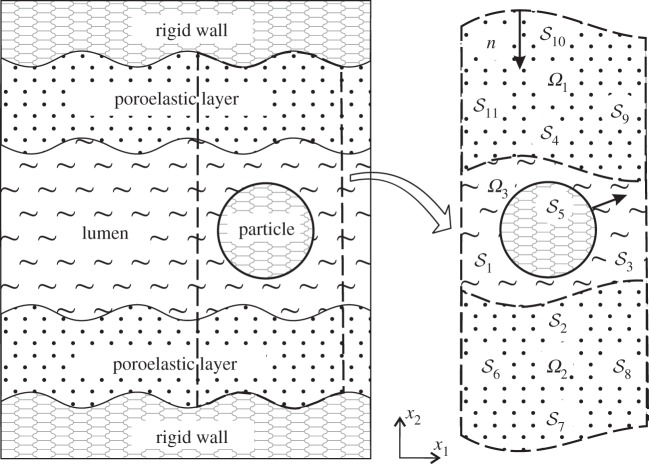
A diagram illustrating two-dimensional geometry of the model, detailing surface labels.

The boundary-integral formulation derived in §2 is very general, applicable in both two and three dimensions. Due to the computational expense, however, we follow [14] and consider a two-dimensional regime, where the vessel is modelled as a channel. Notation specific to this geometry is given in figure 2. We solve the governing integral equations using a BEM scheme, the particulars of which can be found in the electronic supplementary material, S4, alongside validation details.

### (a) Parameter values

We model the elastohydrodynamics of an EGL-coated microvessel containing a rigid cell (such as a white blood cell) with the new boundary-integral formulation. We consider a wavy-walled channel, with top and the bottom channel walls prescribed (in dimensional form) by

3.1 $$S=\left\{\begin{array}{ll} \pm H^{*} & -1-\frac{5}{4}\Lambda_{e}^{*}\leq x_{1}^{*}<-\frac{5}{4}\Lambda_{e}^{*}\\ \eta_{\pm }^{*}(x_{1}^{*}) & -\frac{5}{4}\Lambda_{e}^{*}\leq x_{1}^{*}<-\Lambda_{e}^{*}\\ \pm H^{*}\pm a^{*}\cos\left(\frac{2\pi x_{1}^{*}}{\Lambda_{e}^{*}-\Phi_{\pm }}\right) & -\Lambda_{e}^{*}\leq x_{1}^{*}<\Lambda_{e}^{*}\\ \zeta_{\pm }^{*}(x_{1}^{*}) & +\Lambda_{e}^{*}\leq x_{1}^{*}<\frac{5}{4}\Lambda_{e}^{*}\\ \pm H^{*} & +\frac{5}{4}\Lambda_{e}^{*}\leq x_{1}^{*}\leq 1+\frac{5}{4}\Lambda_{e}^{*}, \end{array}\right.$$

where the positive and negative signs correspond to $S=S_{10}$ and $S=S_{7}$, respectively. The functions η*_±_(*x*_1_), ζ*_±_(*x*_1_) are spline interpolations that guarantee a smooth transition from a straight inlet/outlet to the non-uniform wavy topology (see the electronic supplementary material, S3). Hence, the channel has a mean width of 2H* with wall undulations of amplitude a* and wavelength of Λ*_e_, and we allow for a phase difference between the top and bottom walls of *Φ*_+_=0, *Φ*_−_=*Φ*, similar to [14]. The interfaces between the lumen and the poroelastic layers are located at

3.2 $$S_{4}=S_{10}-\varepsilon^{*}\;\mathrm{and}\;S_{2}=S_{7}+\varepsilon^{*}.$$

Parameter values were chosen to be broadly representative of the movement of a cell in a capillary. Hence, the vessel radius was chosen to be H*=5 μm, representative of a capillary. We consider a spherical particle having radius R*=2.5 μm, which is characteristic of a small lymphocyte. The fluid viscosity is assumed to be that of water *μ*_f_=10^−3^ Pa s. The EGL thickness ε* varies from 0.2 to 0.4 μm up to 1 μm [5]. Although there are currently no direct measurements of hydraulic resistivity within the EGL, estimates are in the range K*=10^10^−10^11^ N s m^−4^ [17]. The shear modulus of the EGL is calculated in [29] to be *ϕ*_s_*μ*_s_=3.5−10 Pa, and it is generally assumed [2] that the EGL has a small solid fraction *ϕ*_s_=0.01. Its Poisson ratio is assumed to be *ν*=0.3. The mean blood velocity in capillaries is *V* *=0.8−1 mm s^−1^ [30]. An endothelial cell has length of approximately Λ*_e_=20−50 μm and height of a*=1−2 μm [14]. Table 1 summarizes these parameters, including values for the non-dimensional quantities which define the dynamics, namely *χ*, *ε*=ε*/H*, *δ*=H*/Λ*_e_, *a*=a*/H* and *R*=R*/H*.

**Table 1.** Typical non-dimensional parameter values for a small lymphocyte negotiating an EGL-lined capillary.

*R*	*χ*	*a*	*ϕ*_s_	*ε*	*δ*	*ν*
0.5	250	0.2	0.01	0.2	0.25	0.3

Accordingly, we consider 10 cases corresponding to different combinations of the position of the particle’s centre, ***x***_c_, and shape of the walls. For each cell position, we also consider two distinct channel shapes: varicose (*Φ*=0) and sinuous (*Φ*=*π*/2) (see table 2 for a summary). The variation of channel height against *x*_1_ for both geometries is shown in the electronic supplementary material, S3.

**Table 2.** Wall shapes and cell positions for the various cases considered.

case	I	II	III	IV	V	VI	VII	VIII	IX	X
***x***_c_	(−2,0)	(−1,0)	(0, 0)	(0, 0.4)	(−2,0)	(−1,0)	(0, 0)	(0, 0.4)	—	—
*Φ*	0	0	0	0	*π*/2	*π*/2	*π*/2	*π*/2	0	*π*/2

In what follows, we present flow fields and associated flow shear stresses, *Γ*_f_=*ϕ*_f_***g***(***x***)⋅***τ*** on the channel walls and *Γ*_c_=***f***(***x***)⋅***τ*** on the cell, corresponding to a varicose geometry, *Φ*=0, and sinuous geometry, *Φ*=*π*/2. In addition, we present predictions for the elastic displacements in the EGL and associated elastic shear stresses on the wall, *Γ*_s_=(*ϕ*_s_(***h***(***x***)−*ϕp****I***⋅***n***)/*ϕ*)⋅***τ*** (here the direction of the tangential vector ***τ*** always coincides with the positive *x*_1_ direction). The total shear stress is *Γ*=*Γ*_f_+*Γ*_s_.

### (b) No cell

**Figure 3.**
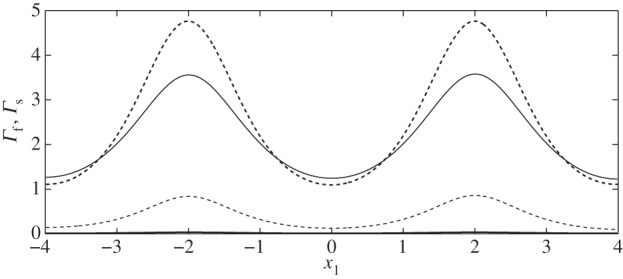
Shear stress distribution on the interface $S_{4}$ and on the solid wall $S_{10}$ in the absence of a cell (case IX): (black solid line) $\Gamma_{s}(S_{4})$; (black dashed line) $\Gamma_{s}(S_{10})$ (elastic stresses); (grey solid line) $\Gamma_{f}(S_{10})$; (grey dashed line) $\Gamma_{f}(S_{4})$ (fluid stresses).

**Figure 4.**
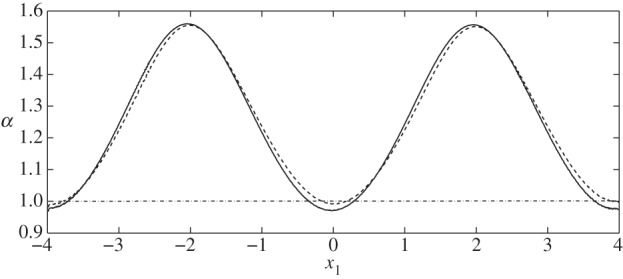
Ratio of the total shear stresses *Γ* exerted on the interface $S_{4}$, compared with that on the wall $S_{10}$, in the absence of a cell. Here, $\alpha =\Gamma (S_{10})/\Gamma (S_{4})$; (solid line) case IX; (dashed line) case X.

Firstly, in figure 3, we compare the stresses on the interface with those on the wall and find that the EGL acts to reduce the FSS (as previously reported), but increases the stress in the solid phase. Combining these two contributions, we observe that in general the total shear stress on the wall is in fact greater than that on the interface. The exception is at the widest section of the vessel, although this is only non-negligible for a varicose vessel (figure 4).

Below, we show the flow fields, stresses (both fluid and elastic) and elastic displacements for both sinuous and varicose microvessels in the absence of the cell. These act as base cases, against which we can compare the cases where a cell is present. We observe that elevated stresses and elastic displacements occur at the geometric constrictions, as expected. Moreover, we note that the magnitude of the shear stress on the wall due to the solid phase dominates over that due to the fluid phase for all cases considered. This appears to support current ideas around the solid phase being the main transducer of mechanical stresses to the underlying endothelial cells [31].

### (c) Varicose geometry with the cell

**Figure 5.**
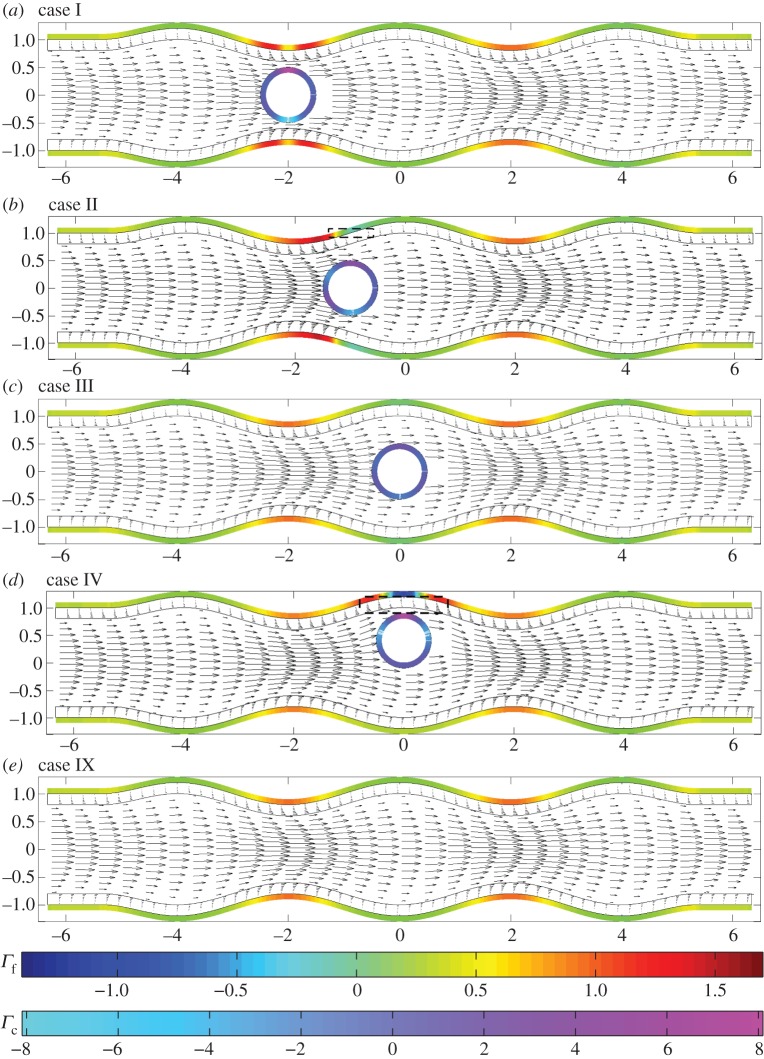
Varicose vessels ((*a*–*d*) cases I–IV and (*e*) IX, respectively) showing flow fields and shear stresses exerted by the flow. The first colour bar indicates the magnitude of the shear stresses on the vessel walls, whereas the scale beneath corresponds to the stresses on the cell. Corresponding translational and angular velocities are: (*a*) ***W***=(0.98,0), *ω*_p_=0; (*b*) ***W***=(0.88,0), *ω*_p_=0; (*c*) ***W***= (0.7,0), *ω*_p_=0; (*d*) ***W***=(0.6,0), *ω*_p_=−0.1. Regions inside dashed boxes in (*b*,*d*) are shown magnified in figure 7.

**Figure 6.**
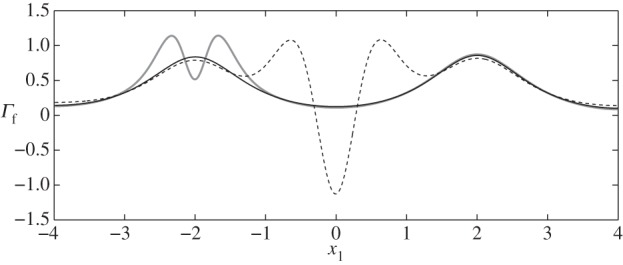
FSS distributions, showing regions of rapid change: (thin black solid line) case IX; (thick grey solid line) case I; (dashed line) case IV.

In figure 5, we examine the flow fields and FSSs for the varicose geometry in the presence of a cell. When the cell is located in the geometric constriction (case I), in figure 5*a*, we observe a local amplification of stresses and flow velocities, over that seen when the cell is absent (figure 5*e*). However, immediately above the cell we observe a reduction in the shear stress. This is highlighted further in figure 6, which shows that the presence of the cell leads to increased wall stress (as compared with the cell-free vessel) immediately upstream and downstream of the cell, but decreased stress directly above the cell (i.e. *x*=−2). When the particle is located on the centreline of the vessel, and in the widest part of the vessel (figure 5*c*), we observe that the influence of the cell upon the FSSs on the wall is fairly minimal.

**Figure 7.**
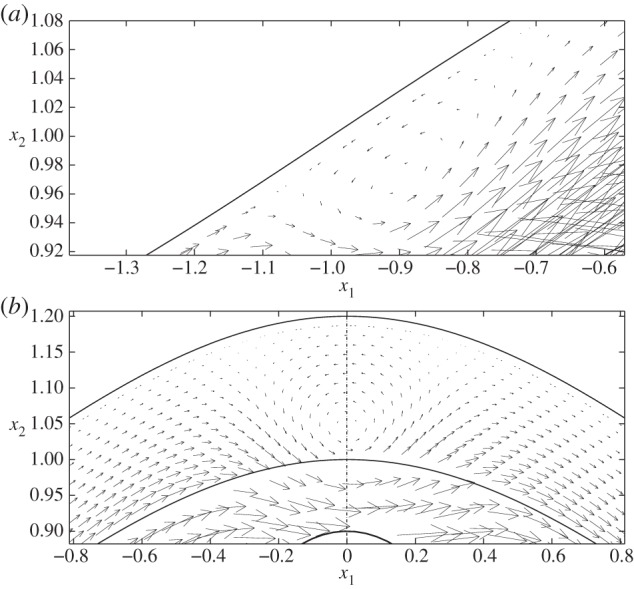
Flow fields in regions of the EGL close to the cell, for (*a*) case II and (*b*) case IV. In both cases, the presence of a vortex is evident. These regions of magnification correspond to the dashed boxes shown in figure 5.

However, when the cell is positioned in the expanding section of the vessel (case II, as shown in figure 5*b*), we again see elevated levels of wall shear in the vicinity of the particle, and a local reduction in shear immediately above and below the cell. In fact, in this scenario, the cell-induced local stress reduction occurs to such an extent that we see a region of negative FSS on the wall. A similar situation occurs when the cell is placed in the widest section of the channel, but close to the upper wall (case IV; figure 6, dashed line). Upon closer examination of the associated flow fields for these two cases (figure 7), we see that this is associated with the presence of a vortex. It was shown in [14] that vortices can appear in a varicose vessel in the absence of a cell, although these were noted to appear in the widest part of the vessel, and for values of *χ*≥1600 which is greater than those considered here. From a physiological standpoint, these flow features are important as they have the capacity to increase the residence time of circulating substances within the EGL. Moreover, the accompanying variations of the shear stress profile could have important implications for mechanotransduction in the microvasculature, as mechanoreceptors located on the surface of the endothelial cells are liable to experience shear stresses exerted by flow within the EGL (fluid component of the total stress).

**Figure 8.**
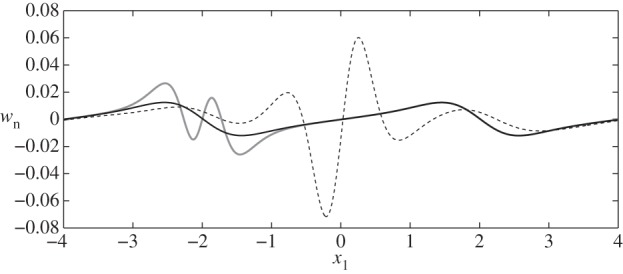
Normal fluid fluxes, *w*_n_, at the interface between core flow and EGL: (thin black solid line) case IX; (thick grey solid line) case I; (dashed line) case IV.

We also analyse the influence of the cell on the normal velocity through the interface, i.e. $w_{n}=\text{ w}\cdot n(S_{4})$, since Wei *et al.* [14] also previously reported a net flux of fluid into the EGL in the absence of a cell. We see in figure 8 that the presence of the particle leads to greater amplitude EGL fluxes (both positive and negative, as compared with the cell-free vessel) in the vicinity of the particle. The highest value of the normal flux occurs for case IV close to the particle ($\max|w_{n}|=0.07$) and the lowest for case X, where there is no cell present ($\max|w_{n}|=0.012$). Although not shown in figure 8, we also note that, for each cell position, phase shifting of the wall does not significantly affect the normal flux distribution.

When we examine the FSSs exerted upon the cell itself in this varicose geometry, we observe that it experiences a much larger shear stress than the solid walls. We also note the degree to which the distribution of stress over the surface changes with cell location in the channel. The cell takes its greatest translational velocity in the constriction, and lower velocities in the widest section of the vessel. As would be expected, for the case of a varicose channel, the angular velocity of the cell is negligible unless moved substantially from the vessel centreline (case IV), where its (non-dimensional) angular velocity is 0.1. In this case, the cell also experiences an up–down asymmetry in shear stress.

**Figure 9.**
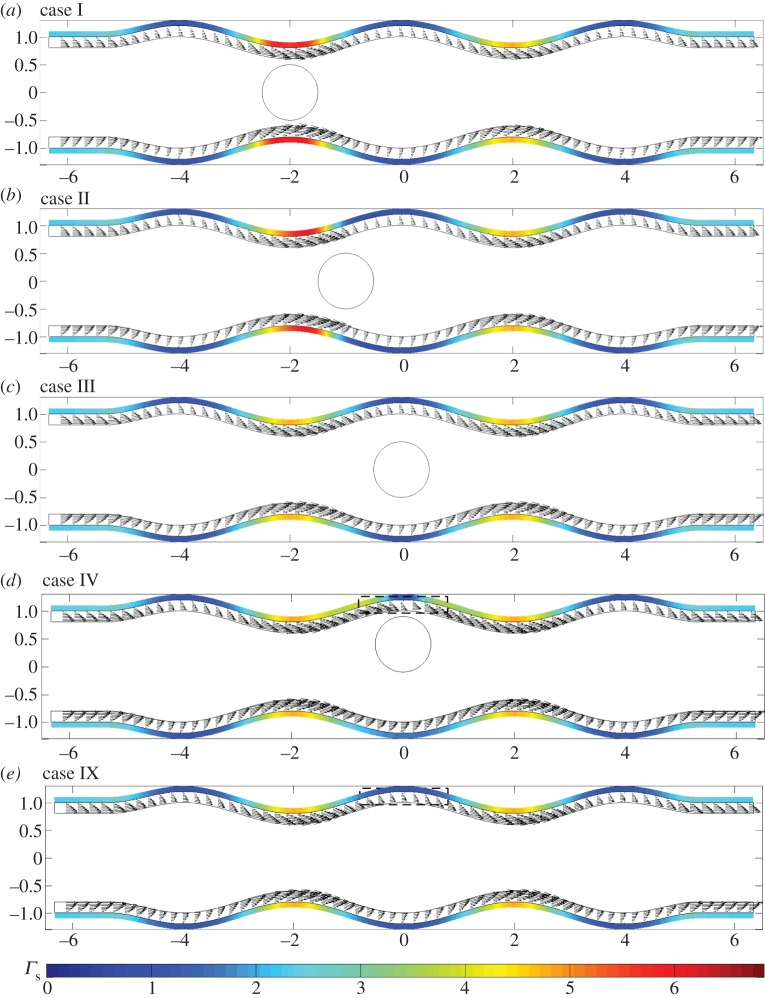
Varicose vessels ((*a*–*d*) cases I–IV and (*e*) IX, respectively) showing elastic displacement vectors and shear stresses exerted by the solid phase, the magnitudes of which are indicated by the colour bars beneath. Regions inside dashed boxes in (*d*,*e*) are shown magnified in figure 10.

**Figure 10.**
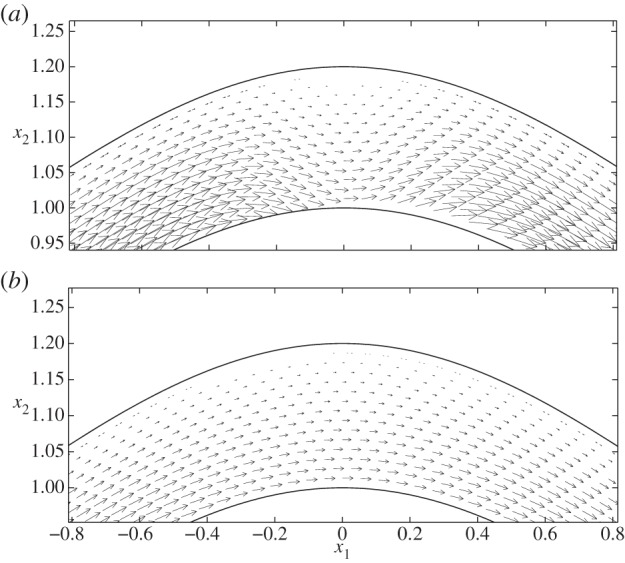
Elastic displacements in the EGL interior: (*a*) case IV and (*b*) case IX. These regions of magnification correspond to the dashed boxes shown in figure 9.

**Figure 11.**
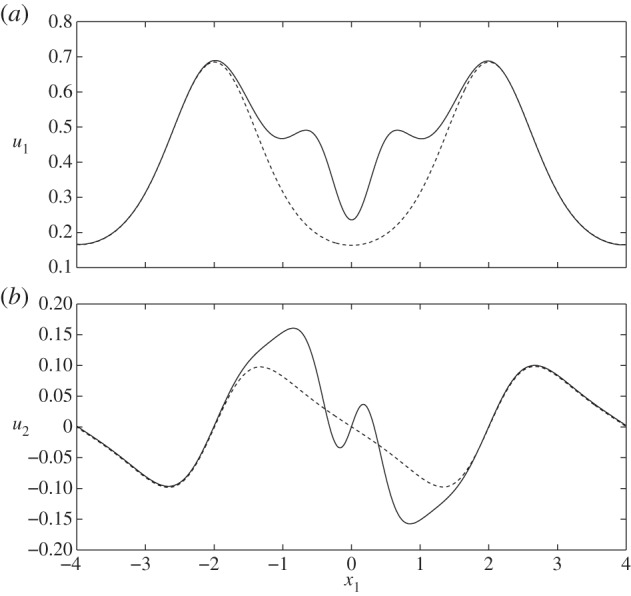
(Non-dimensional) horizontal *u*_1_ (*a*) and vertical *u*_2_ (*b*) elastic displacements on the EGL interface: (solid lines) case IV ; (dashed lines) case IX.

In figure 9, we examine the corresponding displacements and elastic shear stresses. Firstly, as in the no-cell scenario, we see that the elastic stresses are larger in magnitude than the fluid stresses, again offering some support to the notion that a significant proportion of the mechanotransduction in the EGL is performed by the solid phase. We also note that, unlike the FSSs, the elastic shear stresses always remain positive. However, for cases II and IV, where vortex structures were observed in the flow field, we note a corresponding distortion in the elastic displacement field. This is highlighted in figure 10, which compares case IV with the vortex-free case IX. Note that the maximum magnitude of the displacement observed is about 7% of the EGL thickness, and so within the 10% usually accepted for the small strain approximation to be held valid for biological tissue (although a more comprehensive examination of the linear elasticity assumption requires either experimental data or full nonlinear computations; certainly for smaller cells, we expect the linear elasticity assumption to become a better approximation, with the converse being true for larger cells—see the Discussion section for further comments). We can also examine the (non-dimensional) elastic deformations at the interface, and these are shown in figure 11 for the case where no cell is present (case IX), as well as when the cell is close to the upper wall (case IV). We see that the presence of the cell leads to a 65% increase in the displacements induced at the interface.

**Figure 12.**
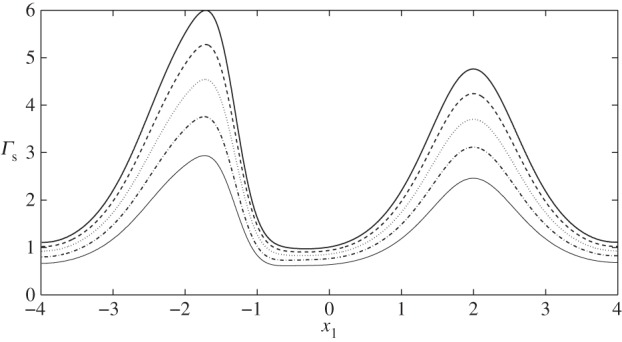
Solid shear stress distribution on the rigid wall corresponding to case II: (black solid line) *ε*=0.2; (dashed line) *ε*=0.175; (dotted line) *ε*=0.15; (dashed-dotted line) *ε*=0.125; (grey solid line) *ε*=0.1.

**Figure 13.**
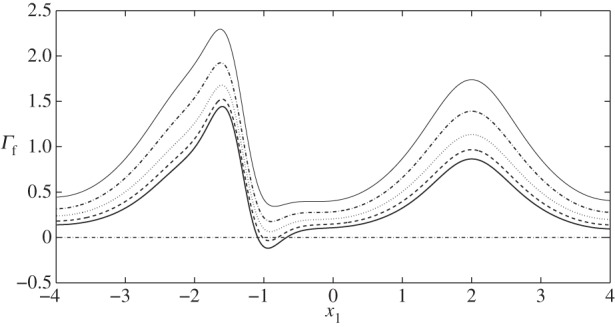
FSS distribution on the rigid wall corresponding to case II: (black solid line) *ε*=0.2; (dashed line) *ε*=0.175; (dotted line) *ε*=0.15; (dashed-dotted line) *ε*=0.125; (grey solid line) *ε*=0.1.

We also briefly investigate the influence of EGL thickness on the stresses exerted on the solid wall. Figures 12 and 13 compare the elastic and fluid stress distributions in case II with *ε*=0.1, *ε*=0.125, *ε*=0.15, *ε*=0.175 and *ε*=0.2. We observe that a reduction in EGL thickness leads to approximately proportional reductions in the elastic stresses exerted upon the wall (e.g. a 50% reduction in EGL thickness leads to an approximately 50% drop in elastic shear). FSSs, however, increase nonlinearly with decreasing layer thickness. Decreasing EGL thickness by 25% leads to an approximately 20% increase in stress while changing it by 50% increases the stress by 60%. In addition, FSS is always positive for *ε*≤0.15, which indicates that for these EGL thicknesses a vortex is not generated.

### (d) Sinuous geometry with the cell

**Figure 14.**
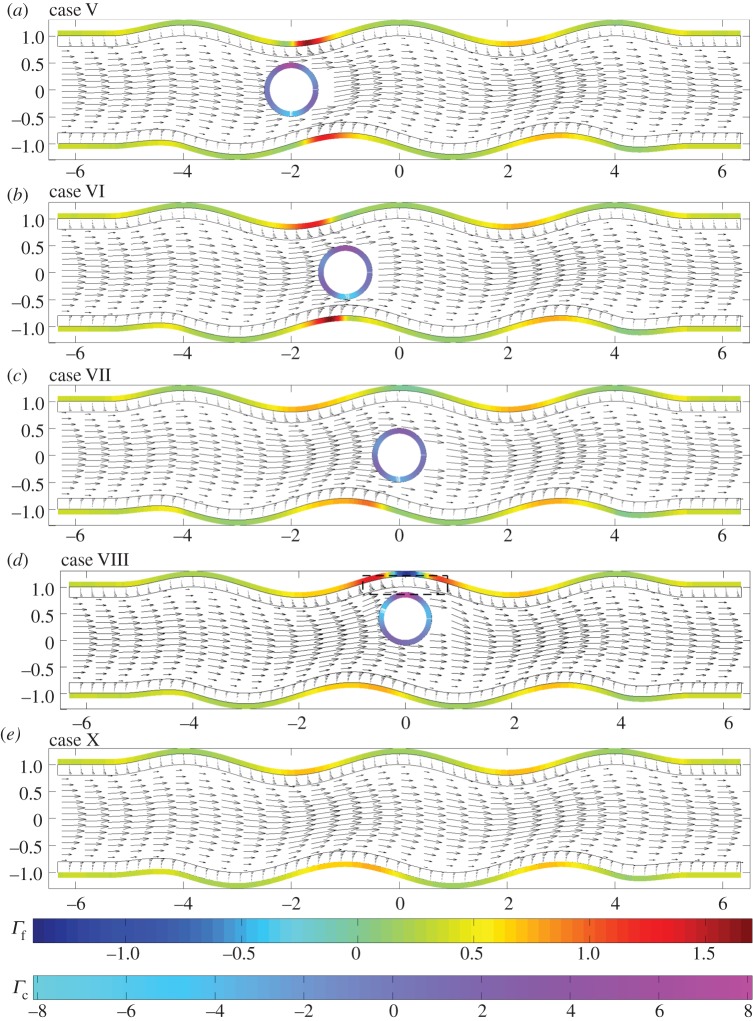
Sinuous vessels ((*a*–*d*) cases V–VIII and (*e*) X, respectively): flow fields and shear stresses exerted by the flow. The top colour bar indicates the magnitude of shear stresses on the wall, whereas the bottom colour bar indicates the magnitude of the cell shear stresses. Corresponding translational and angular velocities are: (*a*) ***W***=(0.92,0.1), *ω*_p_=0; (*b*) ***W***=(0.92,0.1), *ω*_p_=0.1; (*c*) ***W***=(0.82,−0.1), *ω*_p_=0.1; (*d*) ***W***=(0.68,0), *ω*_p_=−0.1. The region inside a dashed box in (*d*) is shown magnified in figure 18.

**Figure 15.**
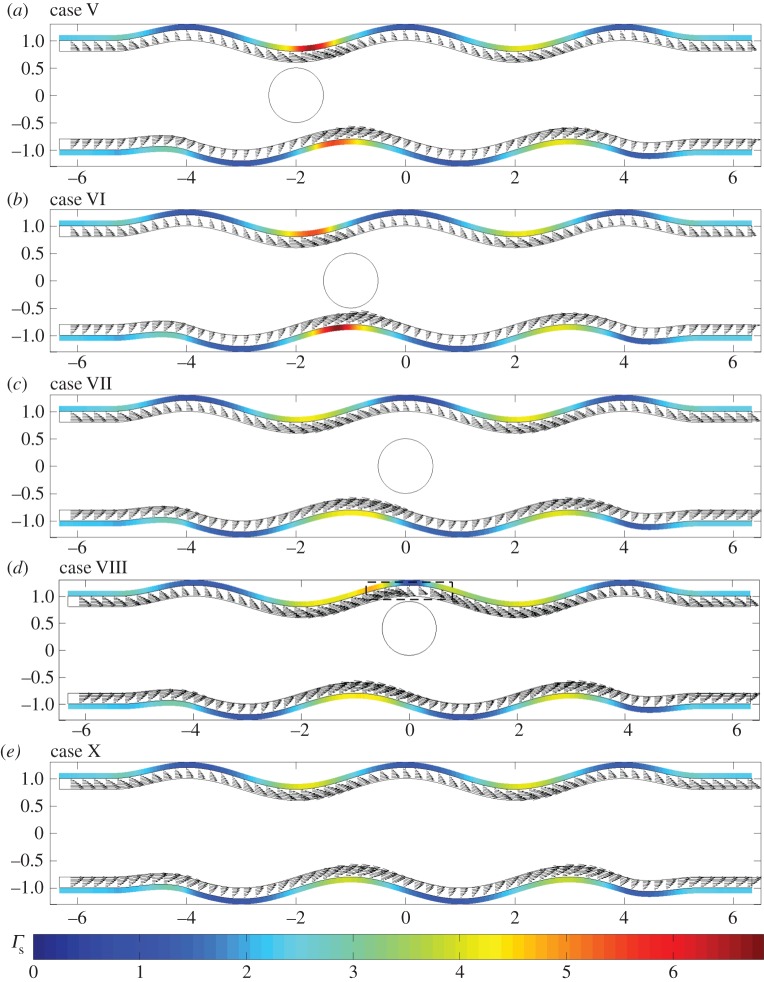
Sinuous vessels ((*a*–*d*) cases V–VIII and (*e*) X, respectively): elastic displacements and shear stresses exerted by the solid phase, the magnitudes of which are indicated by the colour bar. The region inside the dashed box in (*d*) is shown magnified in figure 18.

**Figure 16.**
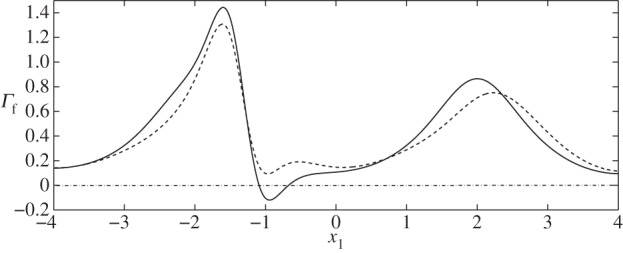
FSS distribution at particle location ***x***_c_=(−1,0), demonstrating the disappearance of negative values when the vessel moves from a varicose shape (case II, solid line) to a sinuous shape (case VI, dashed line).

Let us now briefly examine the changes which occur when the vessel is sinuous (*Φ*=*π*/2). We first note from figures 14 and 15 that the magnitudes of the stresses on the walls and the cell, from both the fluid and solid phases, are comparable with those observed in the varicose case. As the vessel does not expand and contract with downstream distance in the manner of the varicose vessel, the FSSs on the wall have a greater tendency to stay positive. For example, see figure 16, which compares the flow shear stress on the upper wall when ***x***_c_=(−1,0), for both *Φ*=0 (case II) and *Φ*=*π*/2 (case VI). We see that the region of negative FSS disappears.

**Figure 17.**
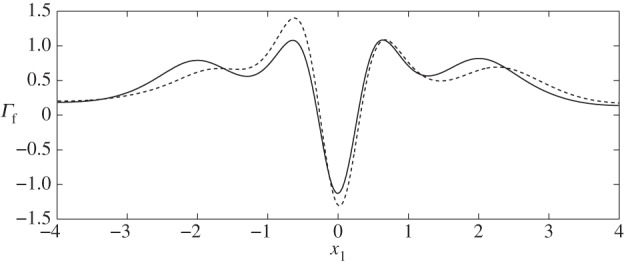
FSS distribution at particle location ***x***_c_=(0,0.4), comparing the differences between the varicose (solid line) and sinuous (dashed line) geometries, i.e. cases IV and VIII.

**Figure 18.**
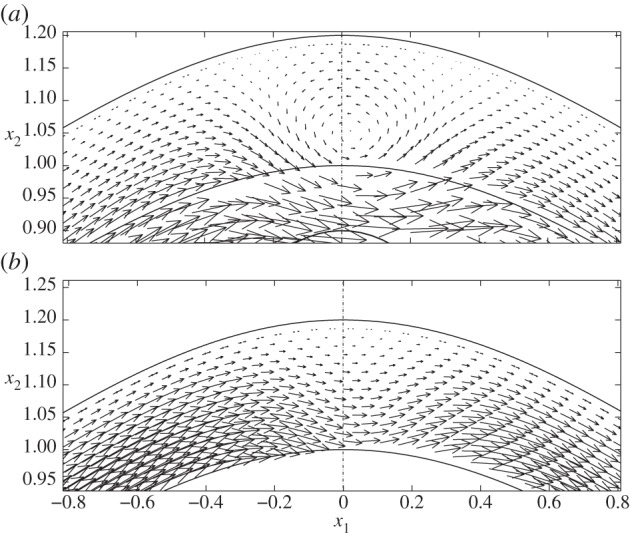
Case VIII: (*a*) flow field, showing the presence of a vortex, and (*b*) associated elastic displacement.

However, if we move the cell sufficiently close to the upper layer (case VIII), figure 14*d* demonstrates that negative FSS on the wall can still occur in the vicinity of the cell and is comparable in magnitude to that observed for the varicose vessel (figure 17). If we examine this region more closely, we see from figure 18 that there is an associated recirculation in the flow field, and corresponding modification of the elastic displacement field. However, this is slightly offset from the cell centre, as compared with that observed in a varicose vessel (cf. figure 7).

In terms of cell mobility, in the sinuous geometry, the cell tends to rotate more and the translational velocity varies within a smaller range (0.68≤***W***≤0.92) than in the varicose geometry (0.6≤***W***≤0.98).

## 4. Discussion

Biphasic mixture theory is widely used to model linearly poroelastic materials. The linearity of the governing equations suggests that a boundary-integral representation is possible. However, the traditional derivations lead to volume integrals of the terms which couple together the solid and fluid phases. By demonstrating how these can be converted into surface integrals, we have been able to derive a true boundary-integral representation for biphasic mixture theory, which we believe will have wide use and applicability.

This has allowed us to examine the elastohydrodynamics of a rigid particle negotiating a poroelastic-lined channel of general shape, as a model for a small lymphocyte negotiating a microvessel coated with an EGL. The EGL is believed to play an important role in transmitting mechanical stresses to the underlying endothelial cells. Indeed, when the EGL is compromised, it has previously been reported that the endothelial cells are less able to align with the flow direction [5]. Numerically solving the boundary-integral representation of the biphasic mixture theory equations, we have been able to examine the effect of vessel shape, and particle position, upon the poroelastic dynamics of the EGL, and motility of the cell. Table 3 summarizes the main findings. Our computations suggest that the shear stresses on the vessel wall consist of significant contributions from the solid phase, and that this contribution increases with increased EGL thickness. This seems to support current theories that suggest that a significant proportion of the mechanical stress applied at the EGL interface is transferred through the solid phase (which is consistent with another one of the EGL’s hypothesized roles in protecting the endothelial cells from excessive FSSs) [5]. We noted that the magnitude of the wall shear stress was a function of EGL thickness. The contribution from the solid phase was seen to decrease (almost linearly) with decreased EGL thickness, in contrast to the contribution from the fluid phase, which increased (nonlinearly) as the EGL became thinner. These findings have potential implications for mechanotransduction through compromised EGLs, and the damage to the underlying endothelial cells which might ensue as a result.

**Table 3.** Summary of findings for the different scenarios considered, including maximum and minimum values of FSSs on the wall ($\Gamma_{f}(S_{10})$), interface ($\Gamma_{f}(S_{4})$) and cell surface ($\Gamma_{f}(S_{5})$). Similarly, elastic stresses on the wall and interface ($\Gamma_{s}(S_{10})$ and $\Gamma_{s}(S_{4})$, respectively). We also summarize the maximum flux of fluid into the EGL (*w*_n_).

	I	II	III	IV	V	VI	VII	VIII	IX	X
$\max\{\Gamma_{f}(S_{10})\}$	1.1	1.4	0.8	1.1	1.6	1.3	0.8	1.4	0.9	0.7
$\max\{\Gamma_{f}(S_{4})\}$	5.2	4.4	3.6	3.7	5.3	4.2	3.2	4.4	3.6	3.2
$\min\{\Gamma_{f}(S_{10})\}$	0.1	−0.1	0.02	−1.1	0.1	0.1	−0.02	−1.3	0.1	0.1
$\min\{\Gamma_{f}(S_{4})\}$	1.2	1.2	1.1	1.2	1.4	1.4	1.0	1.4	1.2	1.2
$\max\{\Gamma_{s}(S_{10})\}$	5.9	6.0	4.8	4.8	6.6	5.5	4.2	4.7	4.8	4.2
$\max\{\Gamma_{s}(S_{4})\}$	0.05	0.04	0.03	0.04	0.05	0.04	0.03	0.04	0.03	0.03
$\min\{\Gamma_{s}(S_{10})\}$	1.1	0.9	0.9	0.8	1.2	1.2	0.9	0.9	1.1	1.2
$\min\{\Gamma_{s}(S_{4})\}$	0.01	0.01	0.01	0.01	0.01	0.01	0.01	0.01	0.01	0.01
$\max\{\Gamma_{c}\}$	7.2	4.2	2.3	7.9	7.3	7.5	3.9	8.2	—	—
$\max\{w_{n}\}$	0.026	0.05	0.013	0.07	0.05	0.04	0.013	0.069	0.013	0.012

In the presence of the rigid cell, we see heightened levels of wall shear from both the fluid and elastic phases. We also find that regions of negative shear stress on the vessel wall can develop in the immediate vicinity of the cell, when it is located relatively close to the EGL. These, in turn, are associated with the presence of recirculating flow regions in the EGL. These recirculating flow regions are seen to be produced more readily in the varicose vessel, where the vessel diameter varies more rapidly. These eddies have previously been reported in the absence of a cell, however only for much higher EGL hydraulic resistivities, and at different locations from those reported here (specifically at the widest section of the microvessel [14]). The regions of recirculating flow could be physiologically important, as they have the potential to increase the residence time of blood components.

Following Wei *et al.* [14], we also examined the flux of fluid into the EGL from the lumen, which potentially has implications for the hypothesized filtering function of the EGL [32]. As in their work, we note the sensitivity of this flux to wall shape, which we observe to be greater in magnitude for a sinuous vessel than for a varicose one. However, we also observe the impact of the cell, the presence of which can lead to an almost fivefold increase in the local flux into the EGL.

In terms of transport of the cell itself, we observe that it generally travels more rapidly through the sinuous vessel than through the varicose one. However, the maximum speed we observe occurs within the constrictions of the varicose vessel. It also exhibits non-negligible angular velocity when close to the EGL, a scenario which occurs more often in the sinuous vessel, where it consequently rotates at more locations along the length of the vessel. These rolling motions are potentially material to lymphocyte recruitment, which forms part of the body’s immune response. We also note that the cell experiences greater shear stresses than the vessel walls, perhaps unsurprisingly given the expected role of the EGL in protecting the endothelial cells from potentially harmful levels of FSS. The cell shear stresses, however, appear to be comparable in magnitude for both shapes considered here, i.e. varicose and sinuous. For computational convenience, we have examined the instantaneous motions of the cells at manually specified positions in the geometry. It would, however, be interesting to track the cell’s trajectory from some starting position. Such simulations would necessarily be very computationally expensive, although they would enable us to determine whether certain configurations are stable or unstable, i.e. determine the extent to which a cell close to the vessel wall remains at this distance.

In keeping with earlier studies, we have used a relatively simple poroelastic, continuum model (i.e. biphasic mixture theory) to encapsulate the elastohydrodynamics of the EGL, and all of its structural complexity. It would be interesting to see whether this particular division of stress from the fluid and solid phases holds under more complex representations of the EGL. It would also be useful to apply our new boundary-integral representation to see how the aforementioned elastohydrodynamics are modified in a three-dimensional setting, using biologically informed vessel geometries, and this is work that we are currently undertaking [33]. We have also assumed small-strain deformations of the EGL, which is legitimate provided that the cells are sufficiently small. However, in order to gauge the range of validity of this linear elasticity assumption, and to model situations where cells are large enough to generate displacements comparable to the EGL thickness, it would be useful to extend the model to incorporate nonlinear elastic effects. On a related theme, another useful extension of the model would be to consider the motion of deformable cells, such as RBCs, through the lumen by adopting a capsule model to capture the finite-strain mechanics of the cell [34].

Finally, there are also additional mechanisms besides elastic forces that are believed to restore the EGL to an equilibrium configuration, following its deformation due to the passage of a cell through a microvessel. These include oncotic processes [17], whereby a difference in the concentration of the plasma proteins in the EGL and lumen plasma generates an oncotic pressure. This pressure difference leads to relaxation of the EGL back to its equilibrium profile. It is also hypothesized that mechano-electrochemical effects can play a similar role [35]. This comes about because the EGL is believed to be hydrated by an electrolytic solution, which contains electrostatically charged macromolecules. The changes in the charge density, which occur when the EGL is compressed, thereby provide another restoring force. It would also be valuable to include both of these effects into our EGL model.

## Supplementary Material

Poroelastohydrodynamics of the EGL: Electronic Supplementary Material

## Data accessibility

The data supporting this article have been uploaded as part of the electronic supplementary material.

## Appendix A

**(a) Pressure forcing terms**

Let us consider the integral corresponding to the *i*th component

A 1 $$\int_{\Omega }p_{,j}(\text{x})S_{ij}(\text{x},{\text{x}}_{0})\;d\Omega =\int_{\Omega }\nabla p\cdot {\bar{\text{S}}}_{i}\;d\Omega ,$$

where vector

A 2 $${\bar{\text{S}}}_{i}=\left\{\begin{array}{ll} -(3-4\nu )\delta_{ij}\ln r+\frac{{\hat{x}}_{i}{\hat{x}}_{j}}{r^{2}},\;j=1,2 & \text{2D,}\\ \frac{(3-4\nu )\delta_{ij}}{r}+\frac{{\hat{x}}_{i}{\hat{x}}_{j}}{r^{3}},\;j=1,2,3 & \text{3D,} \end{array}\right.$$

$\hat{\text{ x}}=({\text{ x}}_{0}-\text{ x})$, and $r=|\hat{\text{ x}}|$. We begin by rewriting ${\bar{\text{ S}}}_{i}$ as

A 3 $${\bar{\text{S}}}_{i}=\nabla ({\hat{x}}_{i}\beta )-4(1-\nu ){\bar{\text{B}}}_{i},$$

where

A 4 $$\left\{\begin{array}{ll} \beta =\ln r,\;{\bar{\text{B}}}_{i}=\delta_{ij}\beta ,\;j=1,2 & \text{2D,}\\ \beta =-\frac{1}{r},\;{\bar{\text{B}}}_{i}=\delta_{ij}\beta ,\;j=1,2,3 & \text{3D.} \end{array}\right.$$

Hence,

A 5 $$\int_{\Omega }\nabla p\cdot {\bar{\text{S}}}_{i}\;d\Omega =\int_{\Omega }\nabla p\cdot \nabla ({\hat{x}}_{i}\beta )\;d\Omega -4(1-\nu )\int_{\Omega }\nabla p\cdot {\bar{\text{B}}}_{i}\;d\Omega .$$

The first area integral in (A 5) can now be converted into a boundary integral by using Green’s first identity together with the fact that pressure is harmonic (∇^2^*p*=0),

A 6 $$\int_{\Omega }\nabla p\cdot \nabla ({\hat{x}}_{i}\beta )\;d\Omega =-\int_{S}{\hat{x}}_{i}\beta \frac{\partial p}{\partial n}\;dl-\int_{\Omega }{\hat{x}}_{i}\beta \nabla^{2}p\;dl=-\int_{S}{\hat{x}}_{i}\beta \frac{\partial p}{\partial n}\;dl.$$

Hence,

A 7 $$\int_{\Omega }\nabla p\cdot {\bar{\text{S}}}_{i}\;d\Omega =-\int_{S}{\hat{x}}_{i}\beta \frac{\partial p}{\partial n}\;dl-4(1-\nu )\int_{\Omega }\nabla p\cdot {\bar{\text{B}}}_{i}\;d\Omega .$$

We also note that

A 8 $$\nabla p\cdot {\bar{\text{B}}}_{i}=\nabla \cdot (p{\bar{\text{B}}}_{i})-p\nabla \cdot {\bar{\text{B}}}_{i}=\nabla \cdot (p{\bar{\text{B}}}_{i})-\frac{1}{2}p\nabla^{2}({\hat{x}}_{i}\beta ).$$

Therefore,

A 9 $$\begin{array}{rl} \int_{\Omega }\nabla p\cdot {\bar{\text{B}}}_{i}\;d\Omega & =\int_{\Omega }\nabla \cdot (p{\bar{\text{B}}}_{i})\;d\Omega -\frac{1}{2}\int_{\Omega }p\nabla^{2}({\hat{x}}_{i}\beta )\;d\Omega \\ & =-\int_{S}p{\bar{\text{B}}}_{i}\cdot \text{n}\;dl+\frac{1}{2}\int_{S}\left(p\frac{\partial ({\hat{x}}_{i}\beta )}{\partial n}-{\hat{x}}_{i}\beta \frac{\partial p}{\partial n}\right)\;dl, \end{array}$$

upon application of the divergence theorem to the first integral, and Green’s second identity to the second integral, together with the fact that pressure is harmonic. Hence, we arrive at the desired representation of the forcing term solely in terms of boundary integrals

A 10 $$\int_{\Omega }\nabla p\cdot {\bar{\text{S}}}_{i}\;d\Omega =(1-2\nu )\int_{S}{\hat{x}}_{i}\beta \frac{\partial p}{\partial n}\;dl+2(1-\nu )\int_{S}2p\beta \delta_{ik}n_{k}-p\frac{\partial ({\hat{x}}_{i}\beta )}{\partial n}\;dl.$$

**(i) Pressure regularization**

In order to regularize the pressure equation (2.27), we first rewrite it as

A 11 $$\begin{array}{rl} c_{f}p({\text{x}}_{0}) & =-\;{⨍\;-}_{S_{m}}g_{i}(\text{x})Q_{i}(\text{x},{\text{x}}_{0})\;ds(\text{x})+\;{⨍\;-}_{S_{m}}\left(w_{i}(\text{x})-w_{i}({\text{x}}_{0}))L_{ik}(\text{x},{\text{x}}_{0})n_{k}(\text{x})\;ds(\text{x}\right)\\ & \;+w_{i}({\text{x}}_{0})\;{⨍\;=}_{S_{m}}L_{ik}(\text{x},{\text{x}}_{0})n_{k}(\text{x})\;ds(\text{x}). \end{array}$$

The second, hyper-singular, integral can now be regularized by considering a Brinkman flow with constant velocity ***V***=***w***(***x***_0_). The corresponding pressure is given by (2.20) as

A 12 ∇p=−χV,

hence *p*=−*χ****V***⋅***x*** and ***g***=−*p****n***. From the boundary-integral representation for pressure (2.27), we obtain

A 13 $$V_{i}{⨍\;=}_{S_{m}}L_{ik}(\text{x},{\text{x}}_{0})n_{k}(\text{x})\;ds(\text{x})=-c_{f}\chi \text{V}\cdot {\text{x}}_{0}+\chi V_{i}{⨍\;-}_{S_{m}}x_{i}Q_{k}(\text{x},{\text{x}}_{0})n_{k}(\text{x})\;ds(\text{x}).$$

We can further simplify the second integral on the right using Green’s third identity

A 14 $${⨍\;-}_{S_{m}}x_{i}Q_{k}(\text{x},{\text{x}}_{0})n_{k}(\text{x})\;ds(\text{x})=-2{⨍\;-}_{S_{m}}x_{i}\left(\frac{\partial \beta }{\partial n}\right)ds(\text{x})=c_{f}{\text{x}}_{0}\cdot {\text{e}}_{i}-2\int_{S_{m}}n_{i}\beta \;ds(\text{x}),$$

where ***e***_*i*_ is the unit vector in the *x*_*i*_ direction. Substituting (A 14) into (A 13) gives us

A 15 $${⨍\;=}_{S_{m}}L_{ik}(\text{x},{\text{x}}_{0})n_{k}(\text{x})\;ds(\text{x})=-2\chi \int_{S_{m}}n_{i}\beta \;ds(\text{x}).$$

Hence,

A 16 $$\begin{array}{rl} c_{f}p({\text{x}}_{0}) & =-{⨍\;-}_{S_{m}}g_{i}(\text{x})Q_{i}(\text{x},{\text{x}}_{0})\;ds(\text{x})\\ & \;+{⨍\;-}_{S_{m}}\left(w_{i}(\text{x})-w_{i}({\text{x}}_{0}))L_{ik}(\text{x},{\text{x}}_{0})n_{k}(\text{x})\;ds(\text{x}\right)-2\chi w_{i}({\text{x}}_{0})\int_{S_{m}}n_{i}\beta \;ds(\text{x}). \end{array}$$

**(b) Complementary flow problem**

**(i) Two-dimensional case**

Let us consider the complementary problem

A 17 $$\nabla \cdot \sigma^{B_{i}}={\text{F}}^{B_{i}},\;i=1,2,$$

where $F_{j}^{B_{i}}=\chi v_{j}^{B_{i}}+S_{ij}$ and ***σ***^*B*_*i*_^ is the Cauchy stress tensors for the fluid phase. Let us consider the case *i*=1, as the solution when *i*=2 is derived similarly. Rewriting (A 17) in terms of components, we have

A 18 $$\nabla^{2}{v_{1}}^{B_{1}}=\frac{\partial p^{B_{1}}}{\partial x_{1}}+\chi {v_{1}}^{B_{1}}-(3-4\nu )\ln r+\frac{{\hat{x}}_{1}^{2}}{r^{2}}$$

and

A 19 $$\nabla^{2}{v_{2}}^{B_{1}}=\frac{\partial p^{B_{1}}}{\partial x_{2}}+\chi {v_{2}}^{B_{1}}+\frac{{\hat{x}}_{1}{\hat{x}}_{2}}{r^{2}},$$

where $\hat{\text{ x}}=({\hat{x}}_{1},{\hat{x}}_{2})=\text{ x}-{\text{ x}}_{0};\;r=|\hat{\text{ x}}|$. Taking the divergence of (A 18) and (A 19), we come to Poisson’s equation for pressure,

A 20 $$\nabla^{2}p^{B_{1}}=2(1-2\nu )\frac{{\hat{x}}_{1}}{r^{2}},$$

which has a solution of the form

A 21 $$p^{B_{1}}=\left(A_{1}+\nu -\frac{1}{2}+\frac{A_{2}}{r^{2}}+(1-2\nu )\ln r\right){\hat{x}}_{1}+\xi (\hat{\text{x}}).$$

Here, *A*_1_,*A*_2_ are constants and $\xi (\hat{\text{ x}})$ is a solution to the Laplace equation ∇^2^*p*=0. There is freedom in the choice of values *A*_1_,*A*_2_ and *ξ*, as, for our purposes, we can choose any boundary conditions to (A 18) and (A 19). Choosing $A_{1}=\frac{1}{2}-\nu ,\;A_{2}=0,\;\xi =0$, we come to the particular solution for pressure,

A 22 $$p^{B_{1}}=(1-2\nu ){\hat{x}}_{1}\ln r.$$

Substituting the expression for pressure (A 22) into (A 18) and (A 19) and solving, we obtain

A 23 v1B1=1−νχ1−2x^12r22ln⁡η−2ln⁡χ+C1I0(η)+C2K0(η)−1+1−4η2+C1I2(η)+C2K2(η)1−2x^12r2

and

A 24 $$v_{2}^{B_{1}}=-2\frac{\left(1-\nu \right)}{\chi }\left(1-\frac{4}{\eta^{2}}+{\tilde{C}}_{1}I_{2}(\eta )+{\tilde{C}}_{2}K_{2}(\eta )\right)\frac{{\hat{x}}_{1}{\hat{x}}_{2}}{r^{2}}.$$

Here, $\eta =\sqrt{\chi }r$, *I*_*α*_(*η*) and *K*_*α*_(*η*) are the modified Bessel functions of the first and second kind, respectively. The solution consists of two parts, namely a general solution of the homogeneous equation and a particular solution. The homogeneous part contains constants that need to be chosen to remove any algebraic singularities at *r*=0. Analysing the small-scale behaviour of *K*_*α*_(*η*) gives [36]

A 25 $$\left.\begin{array}{rl} & K_{0}(\eta )=-\ln\frac{\eta }{2}-\gamma +O(\eta^{2})\approx -\ln\frac{\eta }{2}-\gamma ,\;\eta K_{1}(\eta )=1+O(\eta^{2})\approx 1\\ \;\text{and}\; & K_{2}(\eta )=\frac{2}{\eta^{2}}-\frac{1}{2}+O(\eta^{2})\approx \frac{2}{\eta^{2}}-\frac{1}{2}, \end{array}\right\}$$

*γ*=0.57721… is the Euler–Maschelroni constant. As a consequence, choosing the constant $C_{1}={\tilde{C}}_{1}=0,C_{2}={\tilde{C}}_{2}=2$ leads to the following solution to the complementary problem:

A 26 $$v_{1}^{B_{1}}=\frac{1-\nu }{\chi }\left[2\ln\eta -2\ln\sqrt{\chi }+2K_{0}(\eta )-1+\left(1-\frac{4}{\eta^{2}}+2K_{2}(\eta )\right)\left(1-\frac{2{\hat{x}}_{1}^{2}}{r^{2}}\right)\right]$$

and

A 27 $$v_{2}^{B_{1}}=-2\frac{\left(1-\nu \right)}{\chi }\left(1-\frac{4}{\eta^{2}}+2K_{2}(\eta )\right)\frac{{\hat{x}}_{1}{\hat{x}}_{2}}{r^{2}},$$

which is non-singular as $v_{1}^{B}\to \left(1-\nu \right)(2\ln2-2\ln\sqrt{\chi }-2\gamma -1)/\chi$ and $v_{2}^{B}\to 0$ as r→0.

The final solutions for both terms, ***F***^*B*_1_^ and ***F***^*B*_2_^, can be written as

A 28 $$v_{i}^{B_{i}}=\frac{1-\nu }{\chi }\left(\psi -1+\varphi \left(1-\frac{2{\hat{x}}_{i}^{2}}{r^{2}}\right)\right),\;v_{i}^{B_{j}}=-2(1-\nu )\varphi \frac{{\hat{x}}_{1}{\hat{x}}_{2}}{\chi r^{2}}\;\mathrm{and}\;p^{B_{i}}=(1-2\nu ){\hat{x}}_{i}\ln r,$$

where

$$\begin{array}{rl} \varphi (\eta ) & =1-\frac{4}{\eta^{2}}+2K_{2}(\eta ),\;\psi (\eta )=2\ln\eta -2\ln\sqrt{\chi }+2K_{0}(\eta ),\\ \varphi {\left(\eta \right)}_{x_{i}}^{\prime } & =\frac{\partial \varphi (\eta )/\partial x_{i}}{\partial x_{i}}=\frac{2{\hat{x}}_{i}}{r^{2}}(-\varphi (\eta )+1-\eta K_{1}(\eta )),\;\psi {\left(\eta \right)}_{x_{i}}^{\prime }=\frac{\partial \psi (\eta )/\partial x_{i}}{\partial x_{i}}=\frac{2{\hat{x}}_{i}}{r^{2}}(1-\eta K_{1}(\eta )), \end{array}$$

and the notation *B*_*i*_ relates to the solution corresponding to the ***F***^*B*_*i*_^ term. This has an associated stress tensor (*i*,*j*=1…2, *i*≠*j*)

A 29 σiiBi=−(1−2ν)x^iln⁡r+21−νχ2x^i2r2ψ(η)xi′+φ(η)xi′1−2x^i2r2+φ(η)−4x^ir2+4x^i3r4,

A 30 $$\begin{array}{rl} \sigma_{ji}^{B_{j}} & =\sigma_{ij}^{B_{j}}=\frac{\left(1-\nu \right)}{\chi }\left[\psi {\left(\eta \right)}_{x_{i}}^{\prime }+\varphi {\left(\eta \right)}_{x_{i}}^{\prime }\left(1-\frac{2{\hat{x}}_{j}^{2}}{r^{2}}\right)-2\varphi {\left(\eta \right)}_{x_{j}}^{\prime }\frac{{\hat{x}}_{1}{\hat{x}}_{2}}{r^{2}}+\varphi (\eta )\left(\frac{8{\hat{x}}_{j}^{2}{\hat{x}}_{i}}{r^{4}}-\frac{2{\hat{x}}_{i}}{r^{2}}\right)\right] \end{array}$$

A 31 $$\begin{array}{rl} \;\text{and}\;\sigma_{ii}^{B_{j}} & =-(1-2\nu ){\hat{x}}_{j}\ln r-\frac{4(1-\nu )}{\chi }\left[\varphi {\left(\eta \right)}_{x_{i}}^{\prime }\frac{{\hat{x}}_{1}{\hat{x}}_{2}}{r^{2}}+\varphi (\eta )\left(\frac{{\hat{x}}_{j}}{r^{2}}-\frac{2{\hat{x}}_{j}{\hat{x}}_{i}^{2}}{r^{4}}\right)\right]. \end{array}$$

For small *r*, we have

A 32 $$\varphi (\eta )=O(\eta^{2}),\;\psi (\eta )=2(\ln2-\ln\sqrt{\chi }-\gamma )+O(\eta^{2}),\;\varphi {\left(\eta \right)}_{x_{i}}^{\prime }=O(\eta )\;\mathrm{and}\;\psi {\left(\eta \right)}_{x_{i}}^{\prime }=O(\eta ).$$

It can be seen by substituting (A 32) into (A 28)–(A 31) that the resulting solutions are bounded functions in the neighbourhood of *r*=0. Hence, flow velocities and stresses are non-singular and the integrals (2.33) are proper.

**(ii) Three-dimensional case**

For the complementary problem (A 17) corresponding to the three-dimensional case, we have $F_{j}^{B_{i}}=\chi {\text{ v}}_{j}^{B_{i}}+S_{ij},\;S=(3-4\nu )\delta_{ij}/r+{\hat{x}}_{i}{\hat{x}}_{j}/r^{3},\;i,j=1,2,3.$ Let us consider first the problem corresponding to the vector ***F***^*B*_1_^. The resulting equations are as follows (*i*=1,2,3):

A 33 $$\nabla^{2}v_{i}^{B_{1}}=\frac{\partial p^{B_{1}}}{\partial x_{i}}+\chi v_{1}^{B_{1}}+\frac{\left(3-4\nu \right)}{r}\delta_{i1}+\frac{{\hat{x}}_{1}{\hat{x}}_{i}}{r^{3}}.$$

Taking the divergence of (A 33) and using incompressibility, we obtain

A 34 $$\nabla^{2}p^{B_{1}}=(2-4\nu )\frac{{\hat{x}}_{1}}{r^{3}},$$

which has a solution of the form

A 35 $$p^{B_{1}}=-(1-2\nu )\frac{{\hat{x}}_{1}}{r}.$$

Substituting (A 35) into (A 33), we obtain (*i*=1,2,3)

A 36 $$\nabla^{2}v_{i}^{B_{1}}-\chi v_{i}^{B_{1}}=(2-2\nu )\left(\frac{{\hat{x}}_{1}{\hat{x}}_{i}}{r^{3}}+\frac{\delta_{i1}}{r}\right).$$

Let us first consider equation (A 36) when *i*=1. To solve this equation, we consider the solution in the form

A 37 $$v_{1}^{B_{1}}=(2-2\nu )(v_{1}^{I}+v_{1}^{II}),$$

where

A 38 $$\nabla^{2}v_{1}^{I}-\chi v_{1}^{I}=\frac{4}{3r},\;\nabla^{2}v_{1}^{II}-\chi v_{2}^{II}=\frac{{\hat{x}}_{1}^{2}}{r^{3}}-\frac{1}{3r}.$$

Introducing spherical coordinates ${\hat{x}}_{1}=r\sin\theta \cos\phi ,\;{\hat{x}}_{2}=r\sin\theta \sin\phi ,\;{\hat{x}}_{3}=r\cos\theta$, centred on ***x***_0_, and seeking a solution for $v_{1}^{I}$ that depends upon *r* alone we come to the equations

A 39 $$\frac{\partial^{2}v_{1}^{I}}{\partial r^{2}}+\frac{2}{r}\frac{\partial v_{1}^{I}}{\partial r}-\chi v_{1}^{I}=\frac{4}{3r}$$

and

A 40 $$\begin{array}{r} \frac{\partial^{2}v_{1}^{II}}{\partial r^{2}}+\frac{2}{r}\frac{\partial v_{1}^{II}}{\partial r}+\frac{\cos\theta }{r^{2}\sin\theta }\frac{\partial v_{1}^{II}}{\partial \theta }+\frac{1}{r^{2}}\frac{\partial^{2}v_{1}^{II}}{\partial \theta^{2}}+\frac{1}{r^{2}\sin^{2}\theta }\frac{\partial^{2}v_{1}^{II}}{\partial \phi^{2}}-\chi v_{1}^{II}=\frac{1}{r}\left(\sin^{2}\theta \cos^{2}\phi -\frac{1}{3}\right). \end{array}$$

The solution to equation (A 39) has the form

A 41 $$v_{1}^{I}=C_{1}^{I}\frac{e^{-\sqrt{\chi }}r}{r}+C_{2}^{I}\frac{e^{\sqrt{\chi }r}}{r}-\frac{4}{3\chi r}.$$

The solution to equation (A 40) can be represented in the form

A 42 $$v_{1}^{II}=f(r)(\sin^{2}\theta \cos^{2}\phi -\frac{1}{3}).$$

By substitution of (A 42) into (A 40), we obtain the equation for unknown function *f*(*r*)

A 43 $$f^{\prime \prime }+\frac{2}{r}\;f^{\prime }-\left(\chi +\frac{6}{r^{2}}\right)f=\frac{1}{r},$$

where *f*′=d*f*(*r*)/d*r*. Solving equation (A 43), we find an expression for the function *f*(*r*)

A 44 $$f(r)=C_{1}^{II}\frac{e^{-\sqrt{\chi }}r\left(\chi r^{2}+3\sqrt{\chi }r+3\right)}{r^{3}}+C_{2}^{II}\frac{e^{\sqrt{\chi }r}\left(\chi r^{2}-3\sqrt{\chi }r+3\right)}{r^{3}}-\frac{\left(\chi r^{2}-6\right)}{r^{3}\chi^{2}}.$$

According to (A 37), we can write the solution expressed in Cartesians as

A 45 $$\begin{array}{rl} \frac{1}{2-2\nu }v_{1}^{B_{1}} & =C_{1}^{I}\frac{e^{-\sqrt{\chi }r}}{r}+C_{2}^{I}\frac{e^{\sqrt{\chi }r}}{r}-\frac{4}{3\chi r}+\left(\frac{{\hat{x}}_{1}^{2}}{r^{2}}-\frac{1}{3}\right)\\ & \;\times \left[C_{1}^{II}\frac{e^{-\sqrt{\chi }r}(\chi r^{2}+3\sqrt{\chi }r+3)}{r^{3}}+C_{2}^{II}\frac{e^{\sqrt{\chi }r}(\chi r^{2}-3\sqrt{\chi }r+3)}{r^{3}}-\frac{\chi r^{2}-6}{r^{3}\chi^{2}}\right]. \end{array}$$

In the complementary problem, we do not have any boundary or initial conditions, so the constants to be taken are arbitrary. Let us set $C_{2}^{I}=C_{2}^{II}=0,\;C_{1}^{I}=4/(3\chi )$ and $C_{1}^{II}=-2/\chi^{2}$. This choice is dictated by the fact that the final solution is bounded for large *r* or *χ* and it does not contain a strong singularity as $\sqrt{\chi }r\to 0$. As a result, the final solution can be expressed as

A 46 $$v_{1}^{B_{1}}=\frac{2-2\nu }{\chi }\left[\frac{1}{r}(A(\eta )-1)+\frac{{\hat{x}}_{1}^{2}}{r^{3}}(B(\eta )-1)\right],$$

where ($\eta =\sqrt{\chi }r$)

A 47 $$A(\eta )=2e^{-\eta }\left(1+\frac{1}{\eta }+\frac{1}{\eta^{2}}\right)-\frac{2}{\eta^{2}},\;B(\eta )=-2e^{-\eta }\left(1+\frac{3}{\eta }+\frac{3}{\eta^{2}}\right)+\frac{6}{\eta^{2}}.$$

Similarly, we can find the solution to problems (A 36) when *i*=1,2,

A 48 $$v_{2}^{B_{1}}=\frac{(2-2\nu ){\hat{x}}_{1}{\hat{x}}_{2}}{\chi r^{3}}(B(\eta )-1)\;\mathrm{and}\;v_{3}^{B_{1}}=\frac{(2-2\nu ){\hat{x}}_{1}{\hat{x}}_{3}}{\chi r^{3}}(B(\eta )-1).$$

Summarizing, we can write the solution for the full complementary problem

A 49 $$v_{j}^{B_{i}}=\frac{2-2\nu }{\chi }\left[\frac{1}{r}(A(\eta )-1)\delta_{ij}+\frac{{\hat{x}}_{i}{\hat{x}}_{j}}{r^{3}}(B(\eta )-1)\right].$$
