# Supplementary material for: A boundary-integral representation for biphasic mixture theory, with application to the post-capillary glycocalyx
Source: Proc Math Phys Eng Sci. 2015 Jul 8;471(2179):20140955. doi: 10.1098/rspa.2014.0955 (PMC4528650; doi:10.1098/rspa.2014.0955)
Supplement: Poroelastohydrodynamics of the EGL: Electronic Supplementary Material [file rspa20140955supp1.pdf]

PROCEEDINGS OF THE ROYAL SOCIETY A

rspa.royalsocietypublishing.org

Research

Article submitted to journal

Subject Areas:
Applied Mathematics, Fluid Mechanics, Mathematical Modelling

Keywords:
Biphasic Mixture Theory, Boundary Element Methods, Endothelial Glycocalyx Layer

Author for correspondence:
R. J. Clarke
e-mail: rj.clarke@auckland.ac.nz

Poroelastohydrodynamics of the Endothelial Glycocalyx Layer: Electronic Supplementary Material

P. P. Sumets[1], J. E. Cater[1], D. S. Long[1,2] and R. J. Clarke[1]

[1]Department of Engineering Science, University of Auckland, Auckland, 1142, New Zealand
[2]Auckland Bioengineering Institute, University of Auckland, Auckland, 1142, New Zealand

© The Author(s) Published by the Royal Society. All rights reserved.

1. Fundamental Solutions

In what follows we define the singulariy solutions which appear in the boundary-integral expressions introduced in Section 2 of the main article. Defining

$$\hat{\boldsymbol{x}} = (\hat{x}_1, \hat{x}_2) = \boldsymbol{x} - \boldsymbol{x}_0; \quad r = |\hat{\boldsymbol{x}}|; \quad \frac{\partial}{\partial n} = \boldsymbol{n} \cdot \nabla, \quad \delta_{ij} = \begin{cases} 1, & i = j \\ 0, & i \neq j \end{cases},$$

the Green's tensor, G_{ij} and associated stress tensor, T_{ijk}, for Stokes flow problems are given by [1]

$$G_{ij}(\hat{\boldsymbol{x}}) = \begin{cases} -\delta_{ij} \ln r + \hat{x}_i \hat{x}_j / r^2, & 2D \\ \delta_{ij}/r + \hat{x}_i \hat{x}_j / r^3, & 3D \end{cases}; \quad T_{ijk}(\hat{\boldsymbol{x}}) = \begin{cases} -4\hat{x}_i \hat{x}_j \hat{x}_k / r^4, & 2D \\ -6\hat{x}_i \hat{x}_j \hat{x}_k / r^5, & 3D \end{cases}$$

where δ_{ij} is the Kronecker Delta. The analogous tensors for Brinkman flow are given by [2, 3]

$$M_{ij}(\hat{\boldsymbol{x}}, \chi) = \delta_{ij} A_1(\sqrt{\chi} r) + \frac{\hat{x}_i \hat{x}_j}{r^2} A_2(\sqrt{\chi} r),$$
$$R_{ijk}(\hat{\boldsymbol{x}}, \chi) = -2\left[\delta_{ik} \frac{\hat{x}_j}{r^2} D_1(\sqrt{\chi} r) + \left(\delta_{kj} \frac{\hat{x}_i}{r^2} + \delta_{ij} \frac{\hat{x}_k}{r^2}\right) D_2(\sqrt{\chi} r) + \frac{\hat{x}_i \hat{x}_j \hat{x}_k}{r^4} D_3(\sqrt{\chi} r)\right],$$

where

$$A_1(\eta) = \begin{cases} -2/\eta^2 + 2K_0(\eta) + 2K_1(\eta)/\eta, & 2D \\ \left[2\mathrm{e}^{-\eta}\left(1 + 1/\eta + 1/\eta^2\right) - 2/\eta^2\right]/r, & 3D \end{cases}$$

$$A_2(\eta) = \begin{cases} 4/\eta^2 - 2K_0(\eta) - 4K_1(\eta)/\eta, & 2D \\ \left[-2\mathrm{e}^{-\eta}\left(1 + 3/\eta + 3/\eta^2\right) + 6/\eta^2\right]/r, & 3D \end{cases}$$

$$D_1(\eta) = \begin{cases} 2K_2(\eta) + 1 - 4/\eta^2, & 2D \\ \left[2\mathrm{e}^{-\eta}(1 + 3/\eta + 3/\eta^2) - 6/\eta^2 + 1\right]/r, & 3D \end{cases}$$

$$D_2(\eta) = \begin{cases} 2K_2(\eta) + \eta K_1(\eta) - 4/\eta^2, & 2D \\ \left[\mathrm{e}^{-\eta}(\eta + 3 + 6/\eta + 6/\eta^2) - 6/\eta^2\right]/r, & 3D \end{cases}$$

$$D_3(\eta) = \begin{cases} -8K_2(\eta) - 2\eta K_1(\eta) + 16/\eta^2, & 2D \\ \left[\mathrm{e}^{-\eta}(-2\eta - 12 - 30/\eta - 30/\eta^2) + 30/\eta^2\right]/r, & 3D \end{cases}$$

and $K_\alpha(\eta)$ are the Modified Bessel Functions of the Second Kind. The Kelvin solutions for the Navier equation are given by [4]

$$S_{ij}(\hat{\boldsymbol{x}}) = \begin{cases} -(3 - 4\nu)\delta_{ij} \ln r + \hat{x}_i \hat{x}_j / r^2, & 2D, \\ (3 - 4\nu)\delta_{ij}/r + \hat{x}_i \hat{x}_j / r^3, & 3D, \end{cases}$$

$$K_{ijk}(\hat{\boldsymbol{x}}) = -\frac{2}{r^a}\left[\frac{1 - 2\nu}{r}\left(\hat{x}_k \delta_{ij} + \hat{x}_j \delta_{ik} - \hat{x}_i \delta_{jk}\right) + b\frac{\hat{x}_i \hat{x}_j \hat{x}_k}{r^3}\right],$$

where $a = 1,\ b = 2$ in two dimensions ($a = 2,\ b = 3$ in three dimensions). The singularity solutions that appear within the boundary-integral representation of pressure are given as [2, 3]

$$Q_i(\hat{\boldsymbol{x}}) = \begin{cases} -2\hat{x}_i/r^2, & 2D, \\ -2\hat{x}_i/r^3, & 3D, \end{cases}; \quad L_{ik}(\hat{\boldsymbol{x}}, \chi) = \begin{cases} -2\delta_{ik}\left(2 + \chi r^2 \ln r\right)/r^2 + 8\hat{x}_i \hat{x}_k / r^4, & 2D, \\ -2\left(2 - \chi r^2\right)/r^3 + 12\hat{x}_i \hat{x}_k / r^5, & 3D. \end{cases}$$

2. Inlet and Outlet Conditions

We assume that at the inlet and outlet we have fully developed unidirectional flow. In the lumen this flow consequently takes the simple form (in the two-dimensional case),

$$\boldsymbol{V}_0 = \left(\frac{Ax_2^2}{2} + 1, 0\right), \tag{E.1}$$

where

$$A = \left[\frac{\phi_f}{\chi}\left(\frac{1-\cosh(\varepsilon\sqrt{\chi}) - \sqrt{\chi}(1-\varepsilon)\sinh(\varepsilon\sqrt{\chi})}{\cosh(\varepsilon\sqrt{\chi})}\right) - \frac{(1-\varepsilon)^2}{2}\right]^{-1}.$$

Incoming and outgoing flow in the fluid phase of the poroelastic layer is

$$\boldsymbol{W}_0 = \left(C_1 e^{-\sqrt{\chi}x_2} + C_2 e^{\sqrt{\chi}x_2} - A/\chi, 0\right), \tag{E.2}$$

with

$$C_1 = A\left(\frac{e^{\alpha}}{\alpha^2} - Me^{2\alpha}\right), \quad C_2 = AM, \quad M = \frac{(1-\varepsilon)\,\alpha + e^{\varepsilon\alpha}}{2\alpha^2 e^{\alpha}\cosh(\varepsilon\alpha)}.$$

Here $\alpha = \sqrt{\chi}$ for the upper region and $\alpha = -\sqrt{\chi}$ for the lower region.

Finally, the displacement of the solid phase is given by

$$\boldsymbol{U}_0 = \left(-C_1 e^{-\sqrt{\chi}x_2} - C_2 e^{\sqrt{\chi}x_2} + (A(\phi+1)/2)x_2^2 + Bx_2 + D, 0\right), \tag{E.3}$$

where $B = \sqrt{\chi}(\phi+1)(C_2 e^{\alpha(1-\varepsilon)} - C_1 e^{-\alpha(1-\varepsilon)}) - (\alpha/|\alpha|)A(1-\varepsilon)(\phi+1)$ and $D = C_1 e^{-\alpha} + C_2 e^{\alpha} - A(\phi+1)/2 - (\alpha/|\alpha|)B$. The solution corresponding to a three dimensional unidirectional flow can be found in [5].

3. Channel Geometry

Here we detail the interpolation functions $\eta^*_\pm(x_1), \zeta^*_\pm(x_1)$ used in defining the shape of the channel geometry (3.1). We use a fourth-order polynomial, and prescribe coincidence of the function value, its first and second derivatives with the corresponding values of the cosine function at the junction points $x_1^* = \pm\Lambda_e^*$. At points $x_1^* = \pm 5\Lambda_e^*/4$ we specify that the function is equal to the value of straight section and first derivative of zero. For instance, the function $\eta^*_+(x_1)$ has the form,

$$\eta^*_+(x_1^*) = a{x_1^*}^4 + b{x_1^*}^3 + c{x_1^*}^2 + dx_1^* + e. \tag{E.1}$$

Coefficients are found from the conditions $\eta^*_+(-\frac{5}{4}\Lambda_e^*) = -H^*$, ${\eta^*_+}'(-\frac{5}{4}\Lambda_e^*) = 0$, $\eta^*_+(-\Lambda_e^*) = H^* + a^*$, ${\eta^*_+}'(-\Lambda_e^*) = 0$, ${\eta^*_+}''(-\Lambda_e^*) = -a^* 4\pi^2/{\Lambda_e^*}^2$.

In figure 1 we show the cross-sectional height as a function of x_1 for both sinuous and varicose geometries. This illustrates that for two channels of the same mean height, the varicose geometry undergoes expansions and constrictions that are approximately 40% greater in magnitude than observed in a sinuous geometry.

4. Boundary Element Method Implementation

We numerically solve the governing boundary integral equations detailed in Section 2 by first discretising all surfaces into straight elements of equal length, d, and unknown quantities are assumed constant on these elements. For notational convenience, in what follows we denote the traction $\boldsymbol{f}$ on $\mathcal{S}_l$ by $\boldsymbol{f}_l$, and similarly for other variables. Each element is parameterised by $-1 \le t \le 1$

$$\boldsymbol{x}_l^m(t) = \frac{1-t}{2}\boldsymbol{x}_l^m + \frac{1+t}{2}\boldsymbol{x}_l^{m+1}, \tag{E.1}$$

where $\boldsymbol{x}_l^m$ and $\boldsymbol{x}_l^{m+1}$ denote the start and end positions of the mth element. The length of each element is consequently given by $\mathrm{d}l(\boldsymbol{x}) = |\mathrm{d}\boldsymbol{x}^m/\mathrm{d}t|\ \mathrm{d}t = (d/2)\mathrm{d}t$. The number of elements on surface $\mathcal{S}_l$ is N_l.

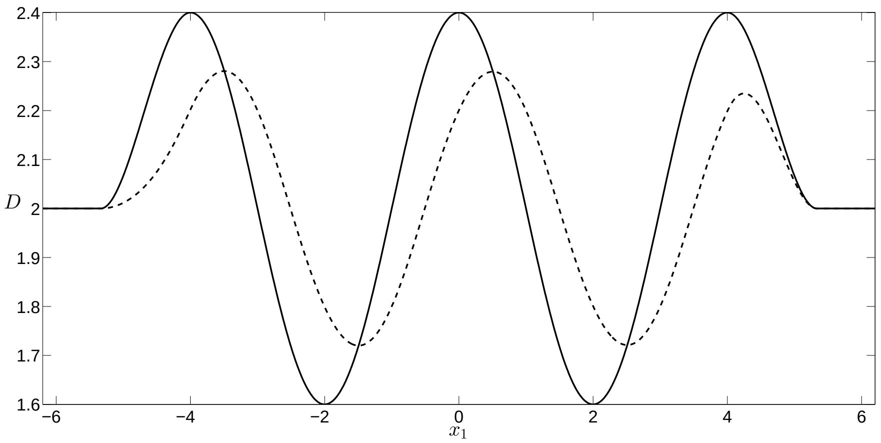

Figure 1. The variation of diameter $D = \mathcal{S}_{10} - \mathcal{S}_7$ for varicose (—) and sinuous (- - -) geometries.

After discretisation, the force free and torque free conditions (2.4) take the form (approximating the integrals by simple Riemann Summations),

$$\frac{d}{2}\sum_{n=1}^{N_5}(f_5^n)_i = 0, \quad \frac{d}{2}\sum_{n=1}^{N_5} Z_{ik}^n (f_5^n)_k = 0, \tag{E.2}$$

$(i = 1, 2)$, where $Z_{ik}^n = \int_{-1}^{1} \epsilon_{ijk}(\boldsymbol{x}_5^n(t) - \boldsymbol{x}_c)_j \,\mathrm{d}t$ with ϵ_{ijk} being the Levi-Civita tensor. The discrete form of the boundary integral formulation for Stokes Flow in the lumen (2.8) becomes $(i, j = 1, 2)$,

$$\sum_{l=1}^{4}\left(\sum_{n=1}^{N_l}(f_l^n)_j G_{ij}^{nm} + \sum_{n=1}^{N_l}(v_l^n)_j T_{ij}^{nm}\right)$$
$$+ \sum_{n=1}^{N_5}(f_5^n)_j G_{ij}^{nm} + W_j \sum_{n=1}^{N_5} T_{ij}^{nm} + \omega_p \sum_{n=1}^{N_5} T_i^{\prime nm} = \begin{cases} c_f(v_e^m)_i, & \boldsymbol{X}_e^m \in S_e \\ c_f(V_p^m)_i, & \boldsymbol{X}_e^m \in S_5 \end{cases} \tag{E.3}$$

$(e = 1 \ldots 4,\ m = 1 \ldots N_e)$ where the collocation point $\boldsymbol{X}_e^m$ is taken to be the midpoint of the mth element, $\boldsymbol{f}_l^n = \boldsymbol{f}(\boldsymbol{X}_l^n)$, $\boldsymbol{v}_l^n = \boldsymbol{v}(\boldsymbol{X}_l^n)$, and $\boldsymbol{V}_p^m = \boldsymbol{V}_p(\boldsymbol{X}_5^m)$. In (E.3), and hereafter, matrix coefficients C_{ij}^{mn} for the single-layer potentials and B_{ij}^{nm} for the double-layer potentials are related to their respective tensors, $\mathcal{C}_{ij}$ and $\mathcal{B}_{ij}$, by

$$C_{ij}^{nm} = -(d/2)\int_{-1}^{1} \mathcal{C}_{ij}\left(\boldsymbol{x}_l^n(t), \boldsymbol{X}_e^m\right)\,\mathrm{d}t, \quad B_{ij}^{nm} = (d/2)\int_{-1}^{1} \mathcal{B}_{ijk}(\boldsymbol{x}_l^n(t), \boldsymbol{X}_e^m) n_k(\boldsymbol{x}^n(t))\,\mathrm{d}t.$$

Also $T_i^{\prime nm} = (d/2)\int_{-1}^{1} \epsilon_{ijk}\,(\boldsymbol{x}_5^n(t) - \boldsymbol{x}_c)_j\,\mathcal{T}_{ijk}\,(\boldsymbol{x}_5^n(t), \boldsymbol{X}_e^m)\,n_k(\boldsymbol{x}^n(t))\,\mathrm{d}t$. Likewise, the discretised form of the boundary integral formulation for Brinkman flow in the poroelastic layer (2.21) becomes,

$$c_f(w_e^m)_i = \sum_{l \in \mathcal{G}}\left(\sum_{n=1}^{N_l}(g_l^n)_j M_{ij}^{nm} + \sum_{n=1}^{N_l}(w_l^n)_j R_{ij}^{nm}\right), \tag{E.4}$$

where $e \in \mathcal{G}$, $m = 1, \ldots, N_e$ and $\mathcal{G} = \{2, 6, 7, 8\}$ for the lower poroelastic layer and $\mathcal{G} = \{4, 9, 10, 11\}$ for the upper layer. Also $\boldsymbol{g}_l^n = \boldsymbol{g}(\boldsymbol{X}_l^n)$, $\boldsymbol{w}_l^n = \boldsymbol{w}(\boldsymbol{X}_l^n)$. The discretised form of the boundary-integral equation relating to the solid phase (2.36) similarly becomes $(m = 1, \ldots, N_e,$

$i, j = 1, 2;\, e, l \in \mathcal{G})$

$$c_s(u_e^m)_i = \sum_{l\in\mathcal{G}} \Bigg(\sum_{n=1}^{N_l} (h_l^n)_j S_{ij}^{nm} + \sum_{n=1}^{N_l} (u_l^n)_j K_{ij}^{nm} + \sum_{n=1}^{N_l} (q_l^n) A^{mn} + \sum_{n=1}^{N_l} (p_l^n) B^{mn} + \sum_{n=1}^{N_l} (w_l^n)_j D_{ij}^{mn} + \sum_{n=1}^{N_l} (g_l^n)_j E_{ij}^{mn} \Bigg), \tag{E.5}$$

where $\boldsymbol{w}_l^n = \boldsymbol{w}(\boldsymbol{X}_l^n)$, $\boldsymbol{h}_l^n = \boldsymbol{h}(\boldsymbol{X}_l^n)$, $p_l^n = p(\boldsymbol{X}_l^n)$, $q_l^n = \partial p(\boldsymbol{X}_l^n)/\partial n$ and

$$A_i^{mn} = -\frac{\phi d(1-2\nu)}{2} \int_{-1}^{1} \left(\boldsymbol{x}^n(t) - \boldsymbol{X}_e^m\right)_i \ln r_{mn}\, \mathrm{d}t,$$

$$B_i^{mn} = -\,\phi d(1-\nu) \int_{-1}^{1} \left(2n_i \ln r_{mn} - \frac{\partial \left((\boldsymbol{x}^n(t) - \boldsymbol{X}_e^m)_i \ln r_{mn} \right)}{\partial n} \right) \mathrm{d}t,$$

$$D_{ij}^{mn} = -\frac{\chi d}{2} \int_{-1}^{1} n_k \sigma_{kj}^{B_i}\, \mathrm{d}t, \quad E_{ij}^{mn} = \frac{\chi d}{2} \int_{-1}^{1} v_j^{B_i}\, \mathrm{d}t.$$

Here $r_{mn} = |\boldsymbol{x}^n(t) - \boldsymbol{X}_e^m|$. Since our elements are straight line segments, contributions from integrals involving strong singularities (e.g. $1/t$) are identically zero (since normals and tangent vectors are perpendicular). Under discretisation, the boundary integral representation of pressure (2.28) takes the form ($m = 1, \ldots, N_e$, $i = 1, 2;\, e, l \in \mathcal{G}$)

$$c_f p_e^m = \sum_{l\in\mathcal{G}} \left(-\sum_{n=1}^{N_l} (g_l^n)_i Q_i^{nm} + \sum_{n=1}^{N_l} \left((w_l^n - w_l^m)_i L_i^{mn} - 2\chi (w_l^m)_i C_i^{mn} \right) \right), \tag{E.6}$$

where

$$C_i^{mn} = \frac{d}{2} \int_{-1}^{1} n_i \ln r_{mn}\, \mathrm{d}t,\; Q_i^{nm} = \frac{d}{2} \int_{-1}^{1} Q_i \left(\boldsymbol{x}^n(t), \boldsymbol{X}_l^m \right) \mathrm{d}t,\; L_i^{nm} = \frac{d}{2} \int_{-1}^{1} L_{ik} \left(\boldsymbol{x}^n(t), \boldsymbol{X}_l^m \right) n_k\, \mathrm{d}t.$$

Finally, Green's Third Identity (2.29) in discretised form becomes

$$\frac{c_f}{2} p_e^m = \sum_{l\in\mathcal{G}} \left(\sum_{n=1}^{N_l} q_l^n H^{mn} + \sum_{n=1}^{N_l} p_l^n J^{mn} \right), \tag{E.7}$$

where $H^{mn} = (d/2)\int_{-1}^{1} \ln r_{mn}\, \mathrm{d}t$ and $J^{mn} = -(d/2)\int_{-1}^{1} \partial(\ln r_{mn})/\partial n\, \mathrm{d}t$. Boundary conditions give us the additional relationships between unknowns. On the impermeable channel walls $\mathcal{S}_{\{7,10\}}$, $\boldsymbol{w}_{\{7,10\}} = \boldsymbol{u}_{\{7,10\}} = \boldsymbol{0}$. On the interfaces, $\mathcal{S}_{\{2,4\}}$, $\boldsymbol{h}_{\{2,4\}} = \phi(\boldsymbol{f} + p\boldsymbol{I}\cdot\boldsymbol{n})_{\{2,4\}}$, $\boldsymbol{g}_{\{2,4\}} = \boldsymbol{f}_{\{2,4\}}$, $\phi_f \boldsymbol{w}_{\{2,4\}} = \boldsymbol{v}_{\{2,4\}}$. On the particle surface, $\mathcal{S}_5$, $\boldsymbol{v}_5 = \boldsymbol{V}_p(\boldsymbol{X}_5^n)$. On the lumen inlet and outlet $\mathcal{S}_{\{1,3\}}$, $\boldsymbol{v}_{\{1,3\}} = \boldsymbol{V}_0(\boldsymbol{X}_{\{1,3\}}^n)$. Finally, on the inlet and outlet for the poroelastic layers, $\mathcal{S}_{\{6,8,11,9\}}$, $\boldsymbol{w}_{\{6,8,11,9\}} = \boldsymbol{V}_0(\boldsymbol{X}_{\{6,8,11,9\}}^n)$, $\boldsymbol{u}_{\{6,8,11,9\}} = \boldsymbol{U}_0(\boldsymbol{X}_{\{6,8,11,9\}}^n)$. Hence, (E.2) - (E.7) provide a linear system which we can be solved to determine the remaining unknown particle translational and angular velocities $\boldsymbol{W}$ and ω_p, interfacial flow velocities $\boldsymbol{w}_2$, $\boldsymbol{w}_4$ and tractions $\boldsymbol{f}_2$ and $\boldsymbol{f}_4$, as well as tractions on the channel walls $\boldsymbol{g}_7$, $\boldsymbol{h}_7$, $\boldsymbol{g}_{10}$ and $\boldsymbol{h}_{10}$.

(a) Numerical Validation and Resources

An element refinement study was conducted to verify convergence of the calculations. The length of segment d was reduced twofold at each refining step j until the desired accuracy was reached, which was evaluated using the relative error estimate $\theta_j = \|\boldsymbol{y}_j - \boldsymbol{y}_{j-1}\|_\infty / \|\boldsymbol{y}_j\|_\infty$, where $\boldsymbol{y}$ is a relevant calculated quantity (e.g. velocity, displacement or traction vectors). In figure 2 we provide

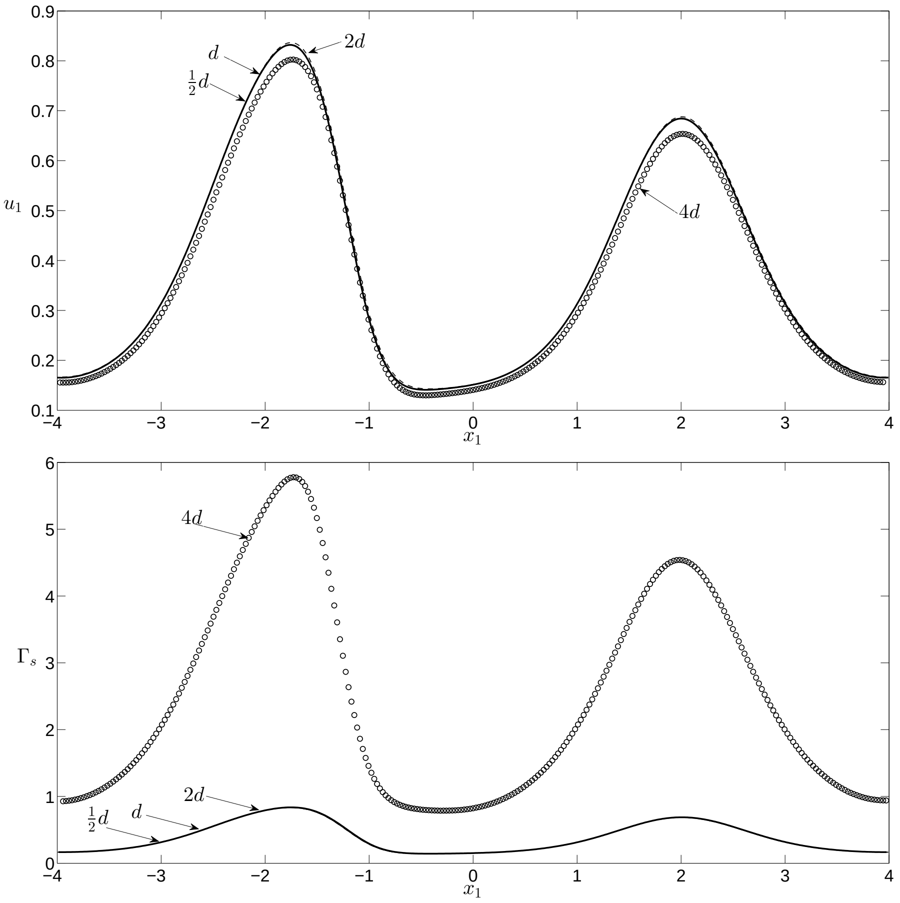

Figure 2. Displacement on the interface (top) and shear stress distribution on the solid wall (bottom) corresponding to Case II and calculated with different element lengths: $\frac{1}{2}d$, d, $2d$ and $4d$, where $d = 6.45 \times 10^{-3}$. Here we have specified a convergence target of $\theta \leq 4 \times 10^{-4}$.

illustrative plots of elastic displacement and shear stress relating to Case II, for differently-sized elements. It both cases, an element size of about 3.2×10^{-3} is sufficient to achieve numerical convergence with $\theta \leq 4 \times 10^{-4}$.

In terms of the required computational resources, construction of the BEM matrices was distributed across 96 parallel processes, with each process allocated 1.5Gb of memory, and taking less than 6 hours in total. The resulting linear system contained 4058 and 4490 unknowns for the EGL and the lumen respectively (as opposed to an analagous volume discretisation scheme, such as the Finite Element Method, where we would have 4058×4058 and 4490×4490 unknowns, respectively). The resulting linear system is solved using LU factorization, as implemented in the LAPACK-based numerics library, with a total solve time of 27 hours for each simulation (i.e. combination of cell position and channel geometry).

(b) Validation

Whilst the fluid component in Biphasic Mixture Theory assumes the standard boundary-integral form for Brinkman flow, the boundary-integral representation of the forced elastic phase (2.36) is new. Hence, in Figure 3 we verify that it is capable of accurately predicting the fluid-forced elastic displacements in the poroelastic lining of straight-walled channel, for which an analytical solution

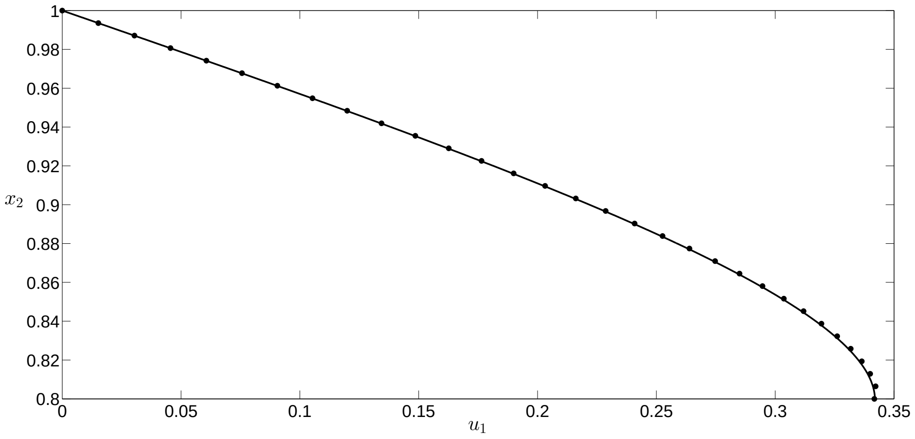

Figure 3. Elastic displacements in the poroelastic lining of a straight-walled channel, as modelled using Biphasic Mixture Theory: (—) analytical solution (E.3); (·) BEM predictions using the new boundary-integral representation (2.36).

exists (E.3). We can see that the agreement is excellent (less than 0.004% difference, which is within the expected discretisation error). In addition, we have compared the results of numerical calculation of the domain integrals and their equivalent boundary integrals given by (2.26) and (2.33). Again, the discrepancy dose not exceed the value of 0.004%.

References

1 Pozrikidis C. Boundary integral and singularity methods for linearized viscous flow. Cambridge University Press; 1992.

2 Kohr M, Sekhar GPR, Wolfgang LW. Boundary integral method for Stokes flow past a porous body. Math Meth Appl Sci. 2008;31(9):1065–1097.

3 Kohr M, Wolfgang LW, Sekhar GPR. Boundary integral equations for two-dimensional low Reynolds number flow past a porous body. Math Meth Appl Sci. 2009;32:922–962.

4 Brebbia CA, Telles JCF, Wrobel LC. Boundary element techniques. Theory and applications in engineering. Springer-Verlag Berlin; 1984.

5 Damiano ER, Duling BR, Ley K, Skalak TC. Axisymmetric pressure-driven flow of rigid pellets through a cylindrical tube lined with a deformable porous wall layer. J Fluid Mech. 1996;314(4):163–189.
